# Targeting PFKFB3 to enhance CDK4/6 inhibitor response in ER+ breast cancer

**DOI:** 10.1186/s13058-026-02336-2

**Published:** 2026-06-25

**Authors:** Sucheta Telang, Brian F Clem, Ariamna A Herrera Miret, Leanne Price, Susan M Dougherty, Anna Schmitz, Xinmin Yin, Xipeng Ma, Xiang Zhang, Jason Chesney, Yoannis Imbert-Fernandez

**Affiliations:** 1 Brown Cancer Center, University of Louisville, Louisville, KY USA; 2 Medicine, University of Louisville, Louisville, KY USA; 3 Departments of Biochemistry and Molecular Genetics, University of Louisville, Louisville, KY USA; 4 San Francisco School of Dentistry, University of California, CA San Francisco, USA; 5 Department of Biology, University of Dayton, Dayton, OH USA; 6 Chemistry, University of Louisville, Louisville, KY USA; 7 Center for Regulatory and Environmental Analytical Metabolomics, University of Louisville, Louisville, KY USA

**Keywords:** CDK4/6 inhibitor, Estrogen receptor, Breast cancer, PFKFB3, Glucose metabolism

## Abstract

**Background:**

Cyclin-dependent kinase 4 and 6 (CDK4/6) inhibitors are widely used in the treatment of estrogen receptor–positive (ER⁺) breast cancer; however, the metabolic adaptations induced by CDK4/6 inhibition remain incompletely defined. In ER⁺ breast cancer, estrogen signaling plays a central role in coordinating cell cycle progression and metabolic programs that support tumor growth. Glycolytic flux is regulated at the level of phosphofructokinase-1 (PFK1) through the inducible enzyme 6-phosphofructo-2-kinase/fructose-2,6-bisphosphatase 3 (PFKFB3), which is transcriptionally regulated by estrogen receptor signaling and has been shown to promote glycolysis and proliferation in ER⁺ breast cancer cells. Yet, how CDK4/6 inhibition intersects with estrogen-regulated glycolytic control to rewire glucose utilization in ER⁺ breast cancer has not been explored.

**Methods:**

Glucose metabolism was assessed using extracellular flux analysis, untargeted metabolomics, and stable isotope tracing with uniformly labeled ^13^C-glucose in ER+ breast cancer cell lines. In vivo metabolic tracing was performed following bolus administration of [U-^13^C]-glucose. The effects of pharmacologic PFKFB3 inhibition, alone and in combination with CDK4/6 inhibitors, were evaluated in vitro and in patient-derived xenograft (PDX) models. Statistical analyses were performed using appropriate tests with correction for multiple comparisons where applicable.

**Results:**

CDK4/6 inhibition increased glycolytic flux, as evidenced by elevated basal and compensatory glycolysis, accumulation of early glycolytic intermediates, and increased ^13^C labeling of fructose 1,6-bisphosphate. PFKFB3 deficiency significantly attenuated the CDK4/6 inhibitor-induced increase in glycolytic flux. Despite increased glycolysis, stable isotope tracing revealed markedly reduced incorporation of glucose-derived carbon into nucleotide biosynthesis and lipid-associated metabolites, consistent with reduced anabolic demand during G1 cell cycle arrest. In vivo glucose tracing demonstrated a dissociation between increased glycolytic flux and downstream biosynthetic utilization. Pharmacologic inhibition of PFKFB3 imposed additional constraints on glucose utilization and significantly enhanced the antitumor efficacy of CDK4/6 inhibition in PDX models.

**Conclusions:**

CDK4/6 inhibition rewires glucose metabolism in ER+ breast cancer by increasing glycolytic flux while limiting downstream glucose utilization, resulting in heightened reliance on regulated glycolytic control to maintain metabolic homeostasis during cell cycle arrest. Disruption of this adaptive metabolic state through PFKFB3 inhibition enhances the antitumor effects of CDK4/6 inhibition and supports the therapeutic potential of targeting glycolytic regulation in combination with CDK4/6 inhibitor-directed therapies.

**Supplementary Information:**

The online version contains supplementary material available at https://doi.org/10.1186/s13058-026-02336-2.

## Background

Estrogen receptor alpha (ERα) drives the progression of ER+/HER2-negative breast cancer and is the molecular basis for current therapeutic approaches [1–3]. Endocrine therapy has steadily improved patient outcomes, evolving from selective estrogen receptor modulators (SERMs), to aromatase inhibitors (AIs), and most recently to selective estrogen receptor degraders (SERDs) [2, 4]. A major therapeutic breakthrough has been the addition of CDK4/6 inhibitors (palbociclib, ribociclib, abemaciclib) to endocrine therapy, which now represents the standard of care for advanced ER+ breast cancer [5–7]. Compared with endocrine therapy alone, CDK4/6 inhibitor combinations nearly double progression-free survival and significantly improve overall survival [8–10].

Mechanistically, CDK4/6 inhibitors suppress CDK4/6-mediated phosphorylation of the retinoblastoma protein (Rb) at Ser 780, thereby maintaining Rb in its active state and inducing G1 cell cycle arrest with consequent inhibition of tumor proliferation [11, 12]. Despite their clinical impact, the cellular programs engaged downstream of Rb activation in response to CDK4/6 inhibition remain incompletely defined. In particular, the mechanisms that determine response, adaptive tumor persistence, and eventual resistance are not fully understood, underscoring the need to identify pathways downstream of CDK4/6 that contribute to therapeutic sensitivity.

Emerging evidence indicates that CDK4/6 inhibitors reprogram cancer cell metabolism, altering glucose utilization, mitochondrial activity, and nucleotide biosynthesis [13–18]. Glycolysis, in particular, has been implicated as a pathway that shapes the response to CDK4/6 inhibition, and inhibition of glucose metabolism has been found to enhance palbociclib efficacy in preclinical models [19]. These findings suggest that metabolic plasticity may enable tumor cell maintenance during treatment, thereby limiting the depth and/or durability of CDK4/6 inhibitor responses.

Among glycolytic regulators, 6-phosphofructo-2-kinase/fructose-2,6-bisphosphatase 3 (PFKFB3) is a key rate-controlling enzyme that governs the synthesis of fructose-2,6-bisphosphate (F2,6BP), a potent allosteric activator of phosphofructokinase 1 (PFK-1) [20, 21]. We previously demonstrated that ER promotes PFKFB3 expression and that PFKFB3 is required for glucose metabolism and proliferative capacity in ER+ breast cancer cells [22]. However, whether CDK4/6 inhibitors engage PFKFB3-dependent metabolic programs, and whether targeting this metabolic node can enhance therapeutic efficacy of CDK4/6 inhibition remains unknown.

Here, we demonstrate that CDK4/6 inhibitors increase PFKFB3 expression and activity in ER+ breast cancer cells, contributing to enhanced glycolytic metabolism. Pharmacological inhibition of PFKFB3 with the selective small-molecule inhibitor PFK-158 attenuates this adaptive metabolic response and potentiates the antitumor effects of CDK4/6 blockade in vitro and in vivo. Together, these findings identify PFKFB3 as an important contributor to adaptive metabolic remodeling following CDK4/6 inhibition that can be therapeutically leveraged to enhance treatment efficacy in ER+ breast cancer.

## Methods

### Cell culture

MCF7 (HTB-22) and T47D (HTB-133) human breast cancer cell lines were obtained from the American Type Culture Collection (ATCC) and maintained at 37 °C with 5% CO_2_. MCF7 cells were cultured in Improved Minimum Essential Medium (IMEM; Corning) supplemented with 10% fetal bovine serum (FBS; Invitrogen). T47D cells were cultured in RPMI-1640 (Gibco) supplemented with 10% FBS (Invitrogen) and human recombinant insulin (Lonza). Cell lines were verified by short tandem repeat (STR) profiling, and all experiments were performed using cells within 15 passages after reconstitution from frozen stocks.

### Drug treatments

Palbociclib (S1116; Selleckchem), ribociclib (S7440; Selleckchem), and PFK-158 (HY-12203; MedChem Express) were used in this study. Palbociclib stock solutions were prepared in sterile water, whereas ribociclib and PFK-158 stock solutions were dissolved in dimethyl sulfoxide (DMSO). All compounds were diluted in complete culture medium to the indicated final concentrations immediately prior to treatment. Vehicle controls (water or DMSO, as appropriate) were included in all experiments.

### RNA extraction and real time PCR

Cells were seeded in six-well plates at a density of 1.5 × 10⁵ cells per well and allowed to adhere overnight prior to treatment. Cells were then treated with the indicated drug concentrations for the specified durations. Total RNA was isolated using the RNeasy Mini Kit (Qiagen) according to the manufacturer’s instructions. Quantitative real-time PCR (qRT–PCR) was performed using TaqMan Gene Expression Assays (Applied Biosystems). PFKFB3 expression was assessed using a validated human TaqMan probe (Hs00190079), with β-actin used as the endogenous control (Hs01060665_g1). Relative gene expression was calculated using the comparative C_T_ (ΔΔC_T_) method as previously described [22] and normalized to the corresponding vehicle controls. Because two independent vehicle control samples were included for each experiment, data were not normalized to a fixed value of 1, allowing for statistical comparison across treatment groups. Statistical significance was determined using two-way analysis of variance (ANOVA). Data represent three biological replicates with two technical replicates per condition.

### Western blot analyses

Cells were harvested and lysed in RIPA buffer supplemented with protease and phosphatase inhibitors (PhosSTOP; Roche) as previously described [22]. Cell lysates were cleared by centrifugation, and protein concentrations were determined using the Pierce BCA Protein Assay Kit (Thermo Fisher Scientific, Waltham, MA, USA). Equal amounts of protein were resolved on 10–12% SDS–polyacrylamide gels and transferred onto Hybond-PVDF membranes (GE Healthcare Life Sciences, Pittsburgh, PA, USA).

Membranes were blocked and incubated overnight at 4 °C with the following primary antibodies: phospho-PFKFB3 (Ser461) (PA5-114619; Invitrogen), PFKFB3 (13763-1-AP; Proteintech), GLUT1 (sc-377228; Santa Cruz Biotechnology), hexokinase II (2867; Cell Signaling Technology), PFK1 (8164; Cell Signaling Technology), pyruvate kinase M2 isoform (4053; Cell Signaling Technology), pyruvate dehydrogenase (PDH; 3205; Cell Signaling Technology), lactate dehydrogenase A (LDHA; 3582; Cell Signaling Technology), and β-actin (A5316; Sigma-Aldrich). Membranes were then incubated with species-specific horseradish peroxidase–conjugated secondary antibodies (anti-mouse or anti-rabbit IgG; Thermo Fisher Scientific, Waltham, MA, USA). Protein bands were visualized using enhanced chemiluminescence (ECL) reagents according to the manufacturer’s instructions (GE Healthcare Life Sciences, Pittsburgh, PA, USA). Scanned band intensities were quantified using UN-SCAN-IT Gel 6.3 software (Silk Scientific, Orem, UT, USA). Densitometric values were normalized as indicated. Quantified values are expressed relative to control. Data represent three biological replicates.

### Glucose uptake

MCF7 and T47D cells were seeded in six-well plates at a density of 2 × 10^5^ cells per well and treated with vehicle or the indicated concentrations of palbociclib or ribociclib for 48 h. Following treatment, cells were washed twice with pre-warmed glucose- and pyruvate-free DMEM (DMEM − Glc/−Pyr/+Gln) supplemented with 10% dialyzed fetal bovine serum and incubated in the same medium for 30 min. Cells were then incubated with 2-[¹⁴C]-deoxy-D-glucose (5 µL; NEC495A050UC, Revvity, Boston, MA, USA) for 15 min at 37 °C in 5% CO₂. After incubation, cells were washed three times with ice-cold glucose-free RPMI medium (− Glc/−Pyr/+Gln), lysed in 0.1% SDS, and lysates were transferred to scintillation vials containing Ultima Gold scintillation fluid (6013321, Revvity, Waltham, MA, USA). Radioactivity was measured using a Tri-Carb 2910 TR liquid scintillation analyzer according to the QuantaSmart™ reference manual.

Because CDK4/6 inhibitor–treated cells undergo G1 cell cycle arrest while continuing to increase in cell size, normalization to metabolic endpoints that scale with cell mass (e.g., protein content or MTT) can confound interpretation of metabolic flux measurements [23]. Therefore, glucose uptake was normalized to cell number. For normalization, parallel plates were seeded and treated identically, and cell numbers were determined at the time of harvest using a hemocytometer. Data represent four biological replicates with two technical replicates per condition.

### Glycolytic rate assay

Cells were seeded in Seahorse XF24 cell culture microplates (Agilent Technologies) at a density of 2.0 × 10⁴ cells per well and allowed to adhere overnight. Cells were then treated with vehicle or the indicated drug conditions for 48 h in complete growth medium. Following treatment, extracellular flux measurements were performed using the Seahorse XF Glycolytic Rate Assay Kit (103344-100; Agilent Technologies, Santa Clara, CA, USA) according to the manufacturer’s instructions and analyzed on an Agilent Seahorse XFe24 Analyzer. Glycolytic rate parameters were calculated using Agilent Seahorse Analytics XF software.

Following completion of the glycolytic rate assay, cell number was determined from the same Seahorse assay wells using the FluoroReporter^®^ Assay (Invitrogen). Cell counts were calculated by interpolation from a standard curve generated under identical assay conditions. Seahorse metabolic flux data were normalized to cell number to assess metabolic activity on a per-cell basis across treatment conditions. This approach is consistent with recommended normalization strategies for extracellular flux measurements, particularly in settings where cellular biomass or composition may be altered [24].

### Measurement of F2,6BP

MCF7 cells were seeded in 10-cm dishes (#430167, Corning) at a density of 1 × 10⁶ cells per dish and treated with vehicle control or 0.5 µM palbociclib or ribociclib for 48 h. At the end of treatment, cells were washed with PBS, harvested, and counted, and equal numbers of cells were used for analysis. F2,6BP levels were measured based on the method described by Van Schaftingen et al. [25], and values were normalized to cell number. Data represent two biological experiments with two technical replicates per condition.

### [U-^13^C]-glucose tracer studies

MCF7 cells were seeded in 10-cm dishes at a density of 3 × 10^6^ cells/dish and treated with vehicle control or 0.5 µM palbociclib for 24 h. Following this initial treatment period, culture medium was replaced with glucose- and pyruvate-free DMEM supplemented with 1 g/L [U-¹³C]-glucose (Cambridge Isotope Laboratories), 10% dialyzed fetal bovine serum, and the corresponding drug treatments and incubated for an additional 24 h. At the end of the labeling period, cells were washed three times with ice-cold 1× phosphate-buffered saline (PBS), and metabolism was rapidly quenched with cold acetonitrile. Metabolites were extracted using an acetonitrile: water: chloroform mixture (2 mL:1 mL:740 µL). Samples were centrifuged at 3,000 × g for 20 min at 4 °C to separate polar, lipid, and cellular debris layers. The remaining cell debris was subjected to a second extraction using chloroform: methanol supplemented with butylated hydroxytoluene (2:1, 1 mM) and centrifuged at 22,000 × g for 20 min at 4 °C. Polar and lipid fractions from both extraction steps were pooled separately. The polar fraction was vacuum-dried by lyophilization and reconstituted in 100 µL of 50% acetonitrile, followed by vigorous vortexing for 3 min. Samples were centrifuged at 14,000 rpm for 20 min at 4 °C, and 80 µL of the clarified supernatant was collected for two-dimensional liquid chromatography–tandem mass spectrometry (2DLC–MS/MS) analysis. Extracted metabolites were subsequently lyophilized and analyzed by Liquid Chromatography Mass Spectrometry (LC/MS) at the Center for Regulatory and Environmental Analytical Metabolomics (CREAM) core facility at the University of Louisville as previously described [16, 26]. Relative abundance levels were calculated by the sum of all isotopologue intensities of the specified metabolite divided by cell number.

### CRISPR/Cas9-mediated generation of PFKFB3-deficient lines

PFKFB3-deficient cell lines were generated using Dharmacon™ Edit-R™ all-in-one lentiviral CRISPR–Cas9 sgRNA constructs (Horizon Inspired Cell Solutions). Two independent sgRNAs targeting human PFKFB3 were used (clone IDs VSGHSOH_37741350 and VSGHSOH_37741355; Cat. No. VSGH12682-16EG5209). A non-targeting sgRNA lentiviral construct (VSGC11964) was used as a control. Cells were seeded in 24-well plates at a density of 1 × 10^5^ cells and transduced at approximately 60% confluency using a multiplicity of infection (MOI) of 0.3. Six hours after transduction, viral supernatant was replaced with complete growth medium. Twenty-four hours later, cells were placed under puromycin selection to enrich for successfully transduced cells. Puromycin-resistant populations were expanded and subsequently passaged for downstream analyses.

### Synergy studies

For drug interaction studies, cells were seeded in 96-well plates at a density of 5,000 cells per well and allowed to adhere overnight. Cells were treated with increasing concentrations of CDK4/6 inhibitors alone or in combination with PFKFB3 knockout using a matrix-based experimental design. Drug concentration ranges were centered around the half-maximal inhibitory concentration (IC₅₀) for each drug and cell line, with concentrations titrated above and below the IC_50_ to generate full dose–response matrices. Cell number was determined using the FluoroReporter^®^ Assay (Invitrogen), with counts interpolated from a standard curve generated under identical assay conditions. Combination data was formatted as concentration–response matrices. Drug interaction analyses were performed using the SynergyFinder web application (SynergyFinder 2.0), and synergy scores were calculated using the Highest Single Agent (HSA) reference model [27]. Combination effects were interpreted based on SynergyFinder-derived HSA synergy scores.

### Mouse studies

All animal experiments were performed in compliance with practices described in the National Institutes of Health Guide for the Care and Use of Laboratory Animals and were approved by the University Committee for Animal Welfare (UCAW) at the University of Louisville. All experiments were conducted in accordance with the approved UCAW protocol and applicable institutional and federal guidelines and regulations.

Female immunodeficient mice (NSG; 6–8 weeks old) were housed under specific pathogen–free conditions. Two estrogen receptor–positive (ER⁺) breast cancer patient-derived xenograft (PDX) models were used. TM00386 was obtained from The Jackson Laboratory PDX Resource and was derived from a primary invasive ER+ ductal carcinoma of the breast. BCM15100 was obtained from the Baylor College of Medicine breast cancer PDX and advanced in vivo models core under a material transfer agreement and was derived from an ER⁺ infiltrating ductal carcinoma.

PDX tumor fragments (~ 2–3 mm³) were orthotopically implanted into the mammary fat pad. To support ER+ tumor growth, mice received 17β-estradiol supplementation via drinking water (2.7 mg/mL; Sigma-Aldrich, Cat. No. E2758) for the duration of the study. Tumor volume was measured every two days using calipers and calculated as (length × width²)/2. Mice were randomized into treatment groups when tumors reached ~ 100–150 mm³. PFK-158·2HCl (Chemgood, Cat. No. PFK158) was formulated in 30% (w/v) Captisol (Selleckchem, Cat. No. S4592) and administered by intraperitoneal injection at a dose of 40 mg/kg on an intermittent schedule (one day on followed by two days off; every 3 days). Palbociclib was formulated in sodium lactate and administered via gavage at a dose of 50 mg/kg once daily. Vehicle-treated control mice received the corresponding formulation without active drug. Animals were monitored regularly for body weight, tumor burden, and overall health. In accordance with University of Louisville UCAW guidelines, the maximal permitted cumulative tumor burden was defined as a tumor diameter greater than 15 mm in an adult mouse, and this limit was not exceeded in any experiment. Animals were euthanized at predefined humane endpoints.

At the experimental endpoint, tumor volume was recorded, followed by nutrient tracing studies and euthanasia by cervical dislocation. Tumors were then excised and processed for downstream analyses, including mass spectrometry analysis and immunohistochemistry.

### *In vivo* [U-¹³C]-glucose infusion and tumor metabolite analysis

At the experimental endpoint, tumor-bearing mice underwent in vivo glucose tracing studies to assess acute glucose utilization. Mice were injected via the tail vein with 80 µL of 25% (w/v) [U-¹³C]-glucose (Cambridge Isotope Laboratories, Cat. No. CLM-1396-1) per injection, administered three times at 15-minute intervals, as previously described [28]. Fifteen minutes after the final injection, mice were euthanized by cervical dislocation in accordance with the approved UCAW protocol. Tumors were rapidly excised, weighed, and flash-frozen for downstream metabolomic analyses. For metabolite extraction, up to 15 mg of pulverized frozen tumor tissue was processed for analysis of polar and lipid metabolites. Metabolites were extracted using acetonitrile/H₂O (60:40, v/v). Extracted metabolites were lyophilized and subsequently analyzed by LC/MS as described for the in vitro stable isotope tracing experiments.

### Immunohistochemistry

Tumor tissues were harvested at the experimental endpoint, fixed in 10% neutral-buffered formalin, and embedded in paraffin. Five-µm sections were cut, mounted on glass slides, deparaffinized in xylene, and rehydrated through graded ethanol to water. Antigen retrieval was performed using citrate buffer in a 2100 Retriever (PickCell Laboratories). Tissue sections were blocked with 10% goat serum for 1 h and then incubated overnight at 4 °C with primary antibodies. Proliferation was assessed using a Ki67 antibody (Abcam, ab16667) at a 1:50 dilution, and PFKFB3 expression was assessed using an anti-PFKFB3 antibody (Proteintech, 13763-1-AP) at a 1:100 dilution. Following primary antibody incubation, sections were washed and incubated with species-appropriate secondary antibodies. Staining was developed with 3,3′-diaminobenzidine tetrahydrochloride (DAB, Vector Laboratories, Burlingame, CA, USA), nuclei were counterstained with hematoxylin (Sigma-Aldrich), and coverslips attached (Permount, Fisher Scientific, Fairlawn, NJ, USA). Stained slides were digitally scanned using a 3DHistech PanDesk slide scanner (Epredia). Ki67 immunohistochemical staining was quantified using QuPath v0.6.0. For each tumor, five independent regions of interest (ROIs) were selected within viable tumor areas. Positive cell detection was performed using identical analysis parameters across all samples, and the percentage of Ki67-positive nuclei was determined for each ROI and averaged to generate a single value for each tumor.

### Statistical analysis

Statistical analyses were carried out using GraphPad Prism (Version 10.6.1 for macOS, GraphPad Software, Boston, Massachusetts USA, www.graphpad.com). All numerical data are reported as mean ± S.D. Grouped analysis was performed using either two-way ANOVA or one-way ANOVA with corrections as indicated. Paired two-tailed Student’s t-tests were used for analyses involving matched or paired samples, as specified in the figure legends. For each experiment, replicates and p-values for all results are listed in their respective figure legends.

## Results

### CDK4/6 inhibition induces PFKFB3 and enhances glycolysis

**Fig. 1:**
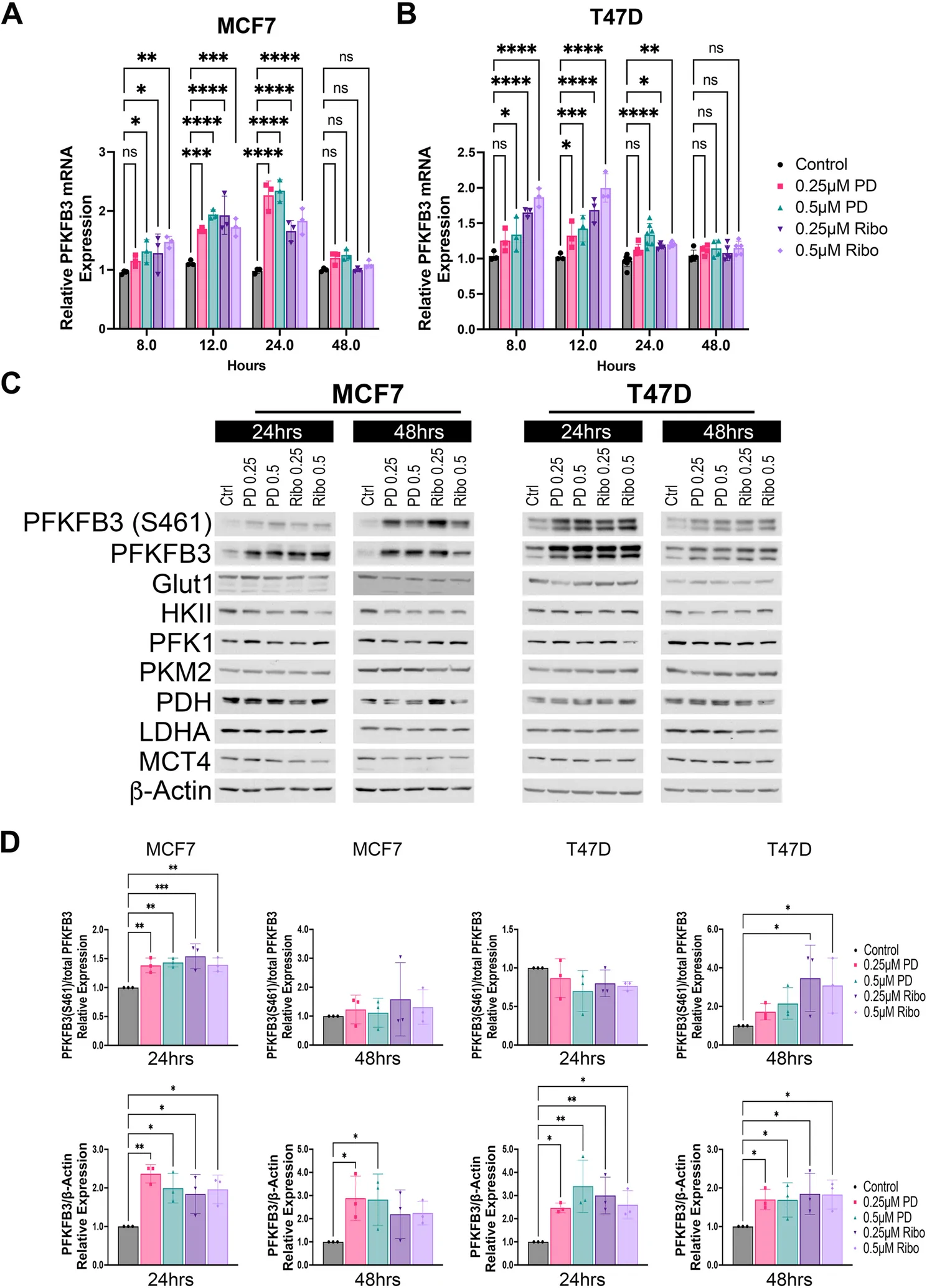
CDK4/6 inhibition induces PFKFB3 expression and phosphorylation in ER⁺ breast cancer cells. **A**–**B** Relative PFKFB3 mRNA expression in MCF7 (**A**) and T47D (**B**) cells treated with palbociclib (PD) or ribociclib (Ribo) at the indicated doses for 8–48 h. Data are shown as mean ± SD of three independent experiments and analyzed by two-way ANOVA with multiple comparisons (*P < 0.05; **P < 0.01; ***P < 0.001; ****P < 0.0001; ns, not significant). **C** Representative immunoblots of total and Ser461-phosphorylated PFKFB3, along with the indicated metabolic proteins in MCF7 and T47D cells treated with palbociclib 0.25 or 0.5 µM (PD) or ribociclib (Ribo) for 24–48 h. **D** Densitometric analysis of total and phospho-PFKFB3 (Ser461) from three biological experiments was performed using UN-SCAN-IT software. Phospho-PFKFB3 (Ser461) was normalized to total PFKFB3, and total PFKFB3 was normalized to β-actin. Data are shown as individual values with SEM indicated and expressed relative to control. Statistical analysis was performed using one-way ANOVA with multiple comparisons

To determine whether metabolic rewiring induced by CDK4/6 inhibition involves PFKFB3, we first examined PFKFB3 mRNA levels in ER⁺ MCF7 and T47D cells exposed to palbociclib and ribociclib for 8–48 h. Both cell lines demonstrated temporal induction of PFKFB3, but with distinct patterns. In MCF7 cells, PFKFB3 mRNA increased rapidly following CDK4/6 inhibition, with significant induction as early as 8 h and maximal expression at 12–24 h prior to returning toward baseline by 48 h (Fig. 1A). In contrast, T47D cells exhibited a more modest response, with peak PFKFB3 expression occurring between 8 and 12 h and a greater magnitude of induction following ribociclib treatment (Fig. 1B). Since PFKFB3 mRNA increased following CDK4/6 inhibition, we next examined whether these transcriptional changes translated into increased protein levels. Endogenous PFKFB3 was markedly increased in both MCF7 and T47D cells after 24–48 h of exposure to palbociclib or ribociclib (Fig. 1C). This increase was accompanied by enhanced phosphorylation of PFKFB3 at Ser461, consistent with activation of its kinase function. Densitometric quantification across independent experiments confirmed a significant increase in total PFKFB3 protein at 24 h in both cell lines, with levels remaining elevated at 48 h in T47D cells but not consistently maintained in MCF7 cells, particularly with ribociclib (Fig. 1D). The ratio of phosphorylated to total PFKFB3 was significantly elevated in MCF7 cells at 24 h across both inhibitors, and in T47D cells more prominently at 48 h, particularly with ribociclib treatment, indicating a cell line– and time-dependent pattern of PFKFB3 activation. In addition to PFKFB3, we examined the expression of key glycolytic enzymes by immunoblotting, with representative blots shown in Fig. 1C. Densitometric analysis revealed that CDK4/6 inhibition did not broadly or consistently alter the expression of these proteins across conditions (Supplementary Fig. 1). GLUT1, HKII, PKM2, PDH, LDHA, and MCT4 showed modest and variable fluctuations that did not reach statistical significance. Notably, PFK1 levels were significantly reduced in T47D cells at 48 h following palbociclib treatment, while no significant changes were observed at earlier timepoints or in MCF7 cells. Taken together, these findings indicate that CDK4/6 inhibition results in a selective activation of PFKFB3, without coordinated upregulation of the broader glycolytic machinery.

### PFKFB3 activation by CDK4/6 inhibition enhances glycolytic flux

**Fig. 2:**
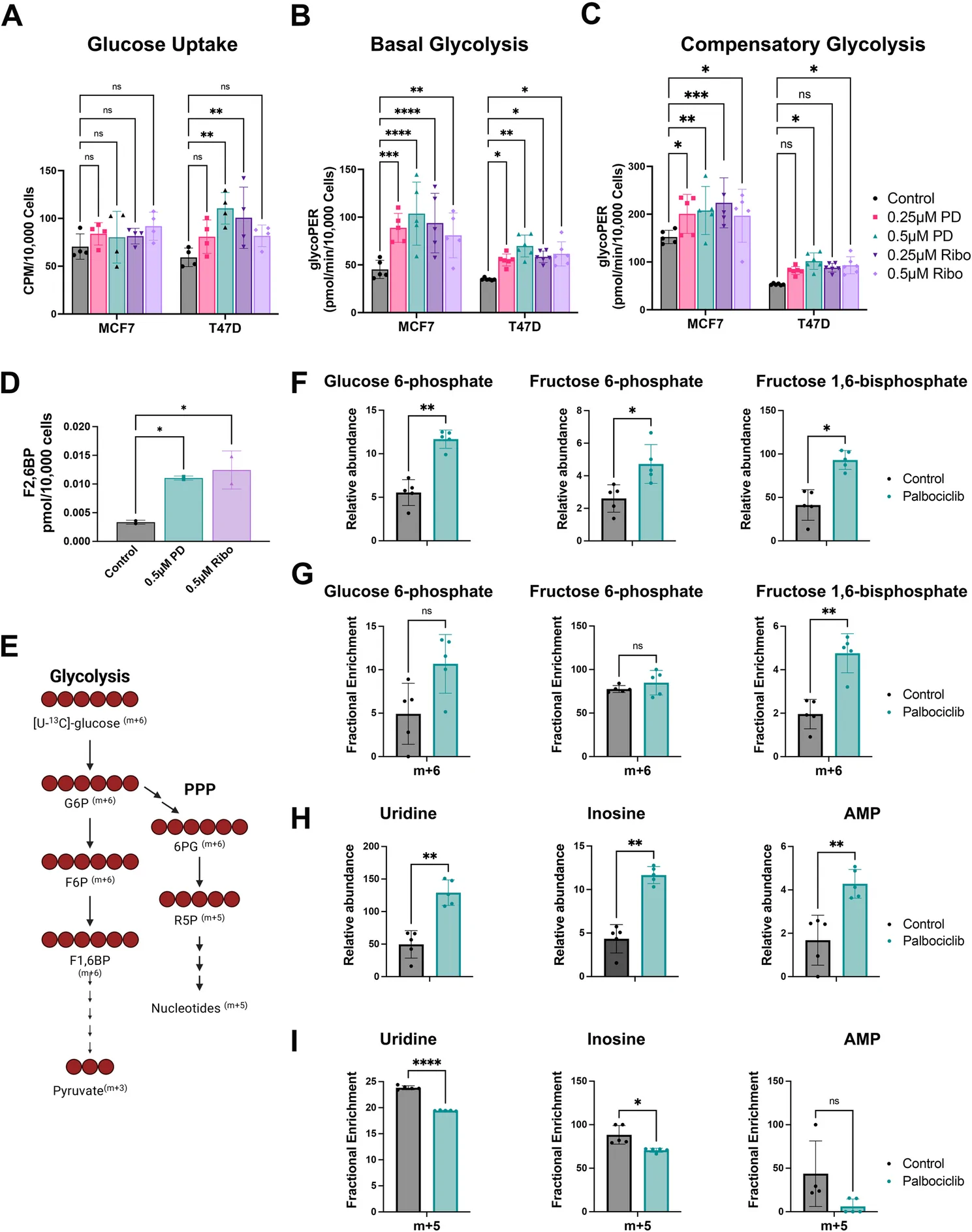
Glucose uptake, glycolytic flux, and metabolic analysis. **A** Glucose uptake in MCF7 and T47D cells treated with palbociclib (PD) or ribociclib (Ribo) at the indicated concentrations. Uptake is expressed as counts per minute (CPM) normalized to cell number. Dots represent biological replicates (n = 4). Statistical analysis was performed using two-way ANOVA with multiple comparisons. **B** Basal glycolysis and **C** compensatory glycolysis measured by Seahorse extracellular flux analysis following treatment with palbociclib or ribociclib. Glycolytic rates are expressed as proton efflux rate (PER) normalized to cell number. Dots represent biological replicates (MCF7, n = 5; T47D, n = 6). Statistical analysis was performed using two-way ANOVA. **D** F2,6BP in MCF7 cells treated with control or 5µM palbociclib or ribociclib for 48 h. Dots represent two biological replicates, each with two technical replicates. Statistical analysis was performed using one-way ANOVA. **E** Schematic of [U-¹³C]-glucose tracing, illustrating carbon flow through glycolysis and diversion into the pentose phosphate pathway (PPP) beginning at glucose-6-phosphate (G6P), leading to ribose-5-phosphate (R5P) and nucleotide synthesis. Expected mass isotopologues (m + 6 for hexose intermediates, m + 5 for ribose) are indicated. **F** Relative abundance of glycolytic intermediates (glucose-6-phosphate, fructose-6-phosphate, and fructose-1,6-bisphosphate) in MCF7 cells treated with control or 0.5µM palbociclib. **G** Fractional enrichment (m + 6) of glycolytic intermediates following [U-¹³C]-glucose tracing in MCF7 cells treated with control or 0.5µM palbociclib. **H** Relative abundance of nucleotide metabolites (uridine, inosine, and AMP) in MCF7 cells treated with control or 0.5µM palbociclib. **I** Fractional enrichment (m + 5) of nucleotide metabolites measured following [U-¹³C]-glucose tracing in MCF7 cells treated with control or 0.5µM palbociclib. Data are presented as mean ± SD of one experiment with 5 technical replicates. Two-way ANOVA with multiple-comparisons testing was used for panels **A**–**C**. Ordinary one-way ANOVA was used for panel **D**. Paired two-tailed Student’s t-tests were used for panels E–H. (*P < 0.05; **P < 0.01; ***P < 0.001; ****P < 0.0001; ns, not significant)

To determine whether increased PFKFB3 expression in response to CDK4/6 inhibition led to functional alterations in glucose catabolism, we next evaluated glucose uptake, glycolytic flux and metabolic fate of glucose following CDK4/6 inhibition. Glucose uptake was modestly increased in T47D cells but remained largely unchanged in MCF7 cells (Fig. 2A). In contrast, basal glycolysis was significantly elevated in both models following CDK4/6 inhibition (Fig. 2B), and this increase was accompanied by a marked rise in compensatory glycolysis, particularly in MCF7 cells (Fig. 2C), indicating enhanced glycolytic flux under CDK4/6 blockade. In line with this, CDK4/6 inhibition increased F2,6BP, the allosteric activator of PFK1 produced by PFKFB3 (Fig. 2D), supporting a role for PFKFB3-mediated activation of glycolytic flux.

To further define how CDK4/6 inhibition alters glucose utilization, we performed stable isotope tracing using [U-¹³C]-glucose, with metabolic pathways and isotopologue labeling patterns summarized in Fig. 2E. This schematic illustrates glucose flux through upper glycolysis to fructose 1,6-bisphosphate (F1,6BP; m + 6), with branch points directing glucose-derived intermediates toward the pentose phosphate pathway (PPP) and downstream nucleotide biosynthesis.

We initially quantified relative metabolite abundance in MCF7 cells following 48 h of palbociclib exposure. Consistent with the activation of the PFKFB3-PFK1 regulatory step, CDK4/6 inhibition significantly increased the abundance of multiple glycolytic intermediates, including glucose 6-phosphate, fructose 6-phosphate, and fructose 1,6-bisphosphate (Fig. 2F). To determine whether these changes reflected increased carbon flux through glycolysis, we next examined isotopologue enrichment from [U-^13^C]-glucose. Although m + 6 fractional enrichment of glucose 6-phosphate and fructose 6-phosphate showed a modest upward trend, these differences were not statistically significant (Fig. 2G). Importantly, m + 6 fructose 1,6-bisphosphate was significantly increased, indicating enhanced glycolytic flux downstream of PFKFB3-PFK1. Downstream intermediates 2PG/3PG exhibited near-complete m + 3 labeling in both conditions, confirming continuous carbon flow through lower glycolysis, while pyruvate pool size increased significantly (~ 2-fold, *p* < 0.001) with maintained high m + 3 enrichment, consistent with enhanced glycolytic flux (Supplemental Fig. 2).

We next examined pathways adjacent to glycolysis, focusing on the pentose phosphate pathway. Palbociclib treatment led to a marked increase in the relative abundance of nucleotide intermediates—including uridine, inosine and AMP (Fig, 2 H). However, incorporation of ^13^C-glucose into the ribose moiety of these nucleotides (m + 5) was significantly reduced in the palbociclib treated samples (Fig. 2I). Given that m + 5 labeling derives from [U-^13^C]-glucose entering the oxidative PPP to generate m + 5 labeled ribose-5-phosphate, the discordance between increased abundance and decreased labeling indicates that nucleotide pools accumulate due to reduced utilization during G1 arrest rather than increased glucose-derived biosynthesis. Taken together, these findings demonstrate that CDK4/6 inhibition selectively enhances glycolytic flux through the PFKFB3-PFK1 node while simultaneously limiting glucose routing into nucleotide synthesis pathways.

### PFKFB3 deficiency attenuates CDK4/6i-induced glycolytic activation

**Fig. 3:**
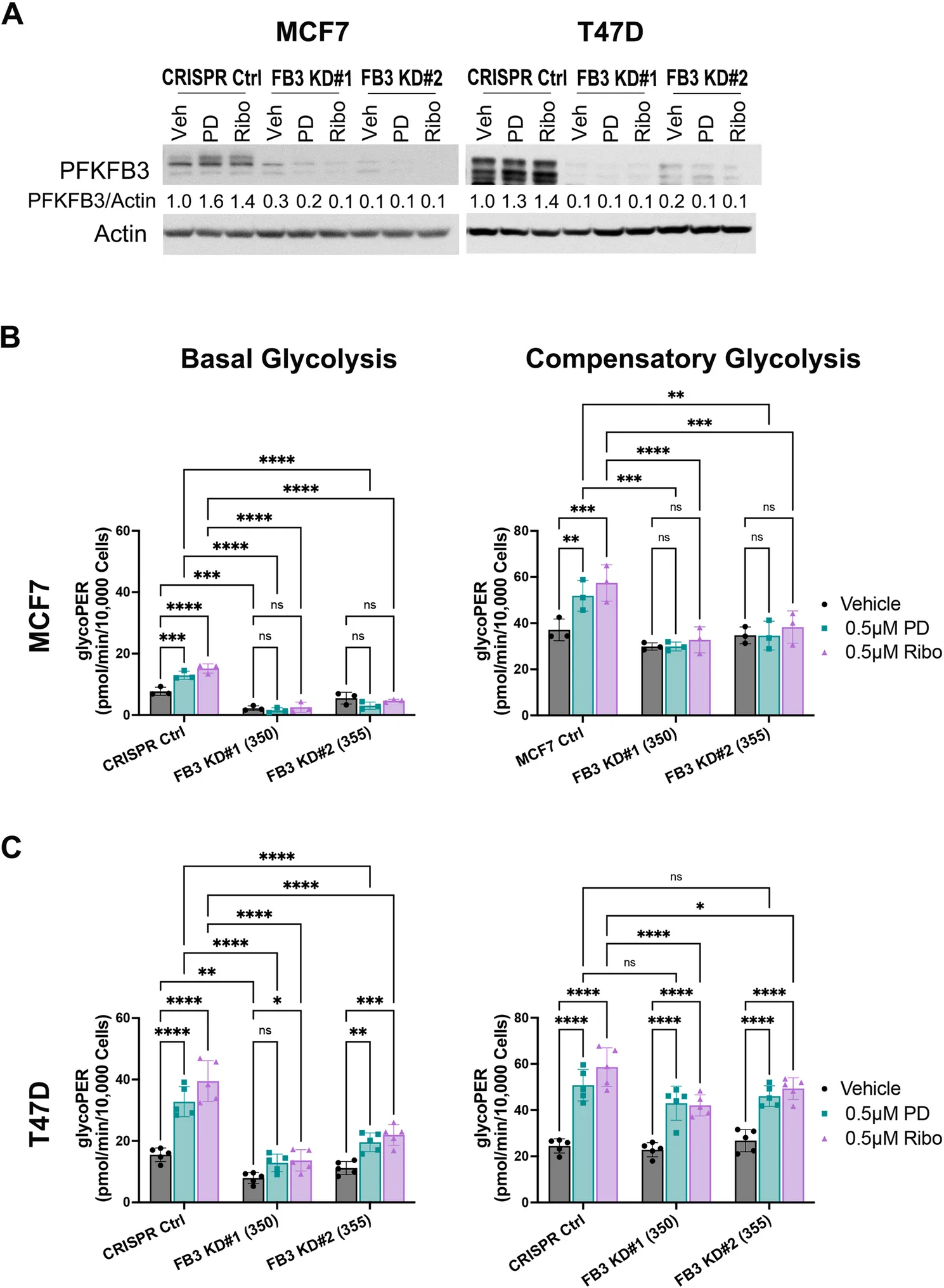
Requirement of PFKFB3 for CDK4/6 inhibitor–induced glycolytic responses.** A** Immunoblot analysis of PFKFB3 expression in MCF7 and T47D CRISPR control cells and PFKFB3-deficient cells generated using two independent sgRNA targeting PFKFB3 (FB3 KD#1 and FB3 KD#2) treated with vehicle, 0.5µM palbociclib (PD), or 0.5µM ribociclib (Ribo). β-Actin was used as a loading control. Representative blots are shown with densitometric quantification normalized to β-actin and expressed relative to vehicle-treated CRISPR control cells. **B**-**C** Basal glycolysis and compensatory glycolysis were measured by Seahorse extracellular flux analysis in MCF7 (top) and T47D (bottom) CRISPR control and PFKFB3-deficient cells following treatment with vehicle, 0.5 µM palbociclib (PD), or 0.5 µM ribociclib (Ribo). Glycolytic rates are expressed as proton efflux rate (PER) normalized to cell number. Data are presented as individual values ± SD of three biological replicates, each performed with three technical replicates. Statistical analysis was performed using two-way ANOVA with multiple comparisons (*P < 0.05; **P < 0.01; ***P < 0.001; ****P < 0.0001; ns, not significant)

To determine whether PFKFB3 contributes to the glycolytic reprogramming induced by CDK4/6 inhibition, we generated two independent CRISPR-edited PFKFB3-deficient cell lines for PFKFB3 in MCF7 and T47D cells. Western blot analysis confirmed marked reduction of PFKFB3 protein, with loss of the palbociclib- and ribociclib-induced upregulation observed in CRISPR control cells (Fig. 3A).

Seahorse glycolytic rate assays revealed that in MCF7 cells, PFKFB3 deficiency abrogated the increase in basal glycolysis induced by CDK4/6 inhibition (Fig. 3B, left). Basal glycolytic rates were also significantly reduced in vehicle-treated PFKFB3-deficient cells compared with CRISPR controls, indicating that PFKFB3 contributes to maintenance basal glycolytic flux in these cells. Compensatory glycolysis was fully suppressed in the PFKFB3-deficient cells following CDK4/6 treatment (Fig. 3B, right), supporting a strong dependence on PFKFB3 for both basal and drug-induced glycolytic capacity in MCF7 cells. In T47D cells, PFKFB3 deficiency also significantly reduced basal and CDK4/6 inhibitor-induced glycolysis; however, the extent of suppression was less pronounced than in MCF7 cells (Fig. 3C, left). CDK4/6 inhibition still induced a modest but significant increase in basal glycolysis in PFKFB3-deficient cells, indicating that glycolytic activation is only partially attenuated in this context. Similarly, compensatory glycolysis in CDK4/6 inhibitor-treated was significantly reduced in PFKFB3-deficient T47D cells but not fully abolished (Fig. 3C, right). Overall, these data indicate that PFKFB3 contributes to the adaptive glycolytic response induced by CDK4/6 inhibition, with a more pronounced effect in MCF7 cells than in T47D.

### PFKFB3 inhibition synergizes with CDK4/6 blockade in ER+ breast cancer cells

**Fig. 4:**
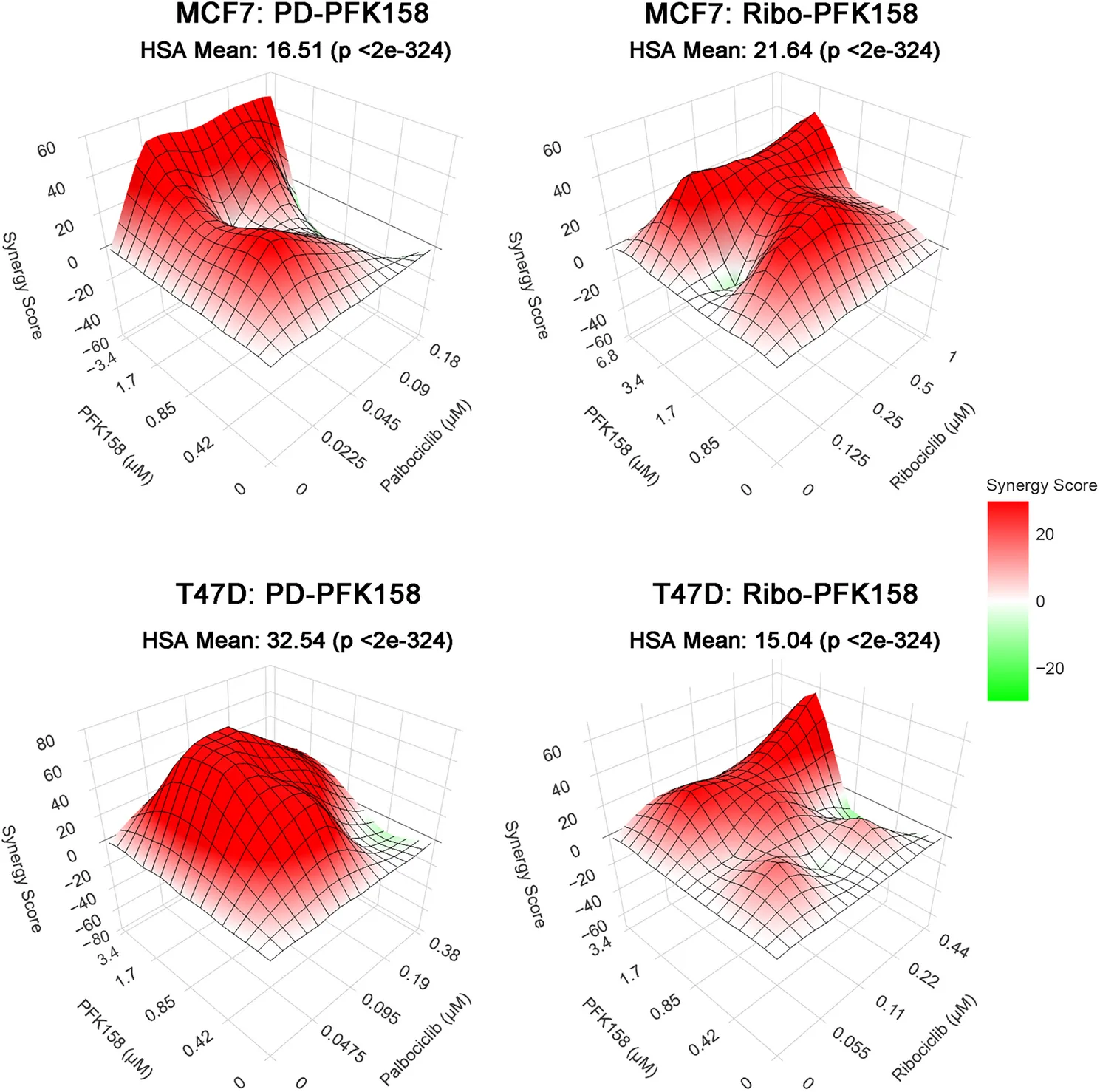
Synergy analysis of CDK4/6 inhibitors combined with PFKFB3 inhibition in ER⁺ breast cancer cells. MCF7 (top) and T47D (bottom) cells were treated with increasing concentrations of CDK4/6 inhibitors in combination with the PFKFB3 inhibitor PFK-158 across the indicated dose matrices. Drug combination response values were used to calculate synergy scores using the SynergyFinder web application with the Highest Single Agent (HSA) model. Three-dimensional response surface plots depict HSA synergy scores across drug combinations, with positive values indicating synergy and negative values indicating antagonism. Mean HSA synergy scores with associated *p*-values for each condition are shown in each plot. Data represent at least three biological replicates; each performed with four technical replicates

Given our findings that CDK4/6 inhibition upregulates glycolysis in a PFKFB3-dependent manner, we hypothesized that dual inhibition of CDK4/6 and PFKFB3 would result in a synergistic anti-proliferative effect. To test this, we performed drug synergy assays in MCF7 and T47D ER+ breast cancer cell lines using a dosing matrix of palbociclib or ribociclib in combination with the PFKFB3 inhibitor PFK-158. Antiproliferative synergy was quantified using the Highest Single Agent (HSA) model, with 3D response surfaces illustrating synergy scores across drug concentration matrices (Fig. 4). Positive scores (red) indicate synergy, while negative scores (green) reflect antagonism. In MCF7 cells, both palbociclib-PFK-158 and ribociclib-PFK-158 combinations demonstrated moderate synergy, with HSA mean scores of 16.51 and 21.64, respectively. In T47D cells, synergy was more pronounced; palbociclib-PFK-158 combination produced a strong synergistic interaction (HSA mean = 32.54), while ribociclib-PFK-158 also showed significant synergy, though to a lesser extent (HSA mean = 15.04). These results demonstrate that combined inhibition of CDK4/6 and PFKFB3 is more effective than either drug alone and support a therapeutic strategy targeting both cell cycle progression and glycolytic adaptation in ER+ breast cancer.

### Dual CDK4/6 and PFKFB3 inhibition enhances antitumor activity in ER + PDX models

**Fig. 5:**
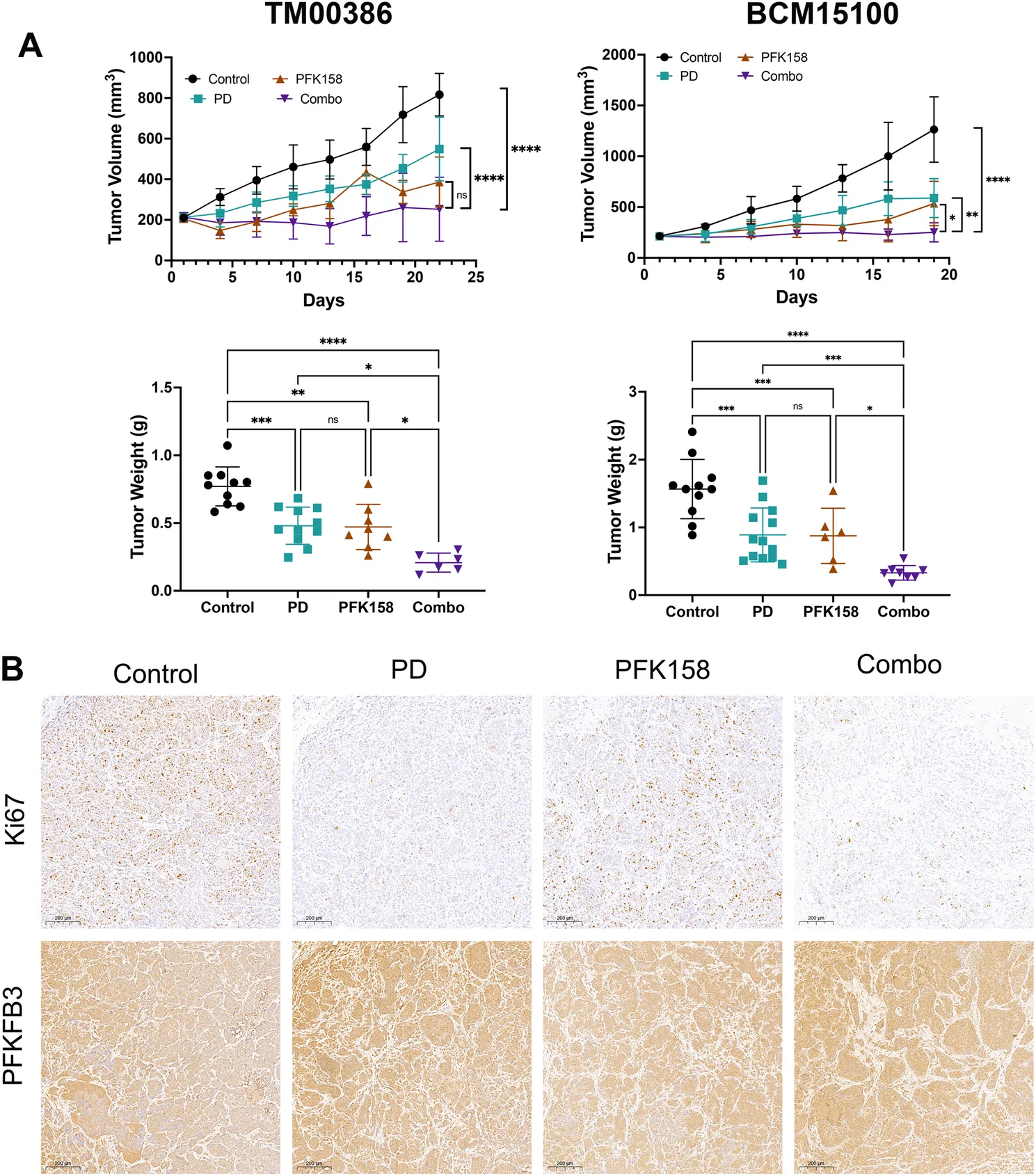
Combined CDK4/6 and PFKFB3 inhibition reduces tumor growth in ER⁺ PDX breast cancer models. **A** Tumor growth curves (top) and tumor weights at endpoint (bottom) from ER⁺ PDX models TM00386 (left) and BCM15100 (right). Mice were treated with vehicle control, palbociclib (50 mg/kg), PFK-158 (40 mg/kg), or the combination, administered daily for palbociclib and every 3 days for PFK-158. Tumor volume was measured over the indicated time course, and tumor weights were recorded at the study endpoint. Data are presented as mean ± SD for tumor volume and as individual values with mean ± SD for tumor weight. Statistical analysis was performed using two-way ANOVA with multiple comparisons testing. **B** Representative immunohistochemical staining of tumor sections from PDX models treated with vehicle control, palbociclib, PFK-158, or the combination. Sections were stained for Ki67 and PFKFB3. Images shown are representative of tumors from BCM15100-treated animals

We evaluated the therapeutic efficacy of combined CDK4/6 and PFKFB3 inhibition in vivo using two patient-derived xenograft (PDX) models of ER+ breast cancer. Mice bearing TM00386 or BCM15100 tumors were treated with vehicle, palbociclib (50 mg/kg), PFK-158 (40 mg/kg), or their combination as described Methods. In both PDX models, palbociclib and PFK-158 monotherapies exerted tumor growth suppression as indicated by tumor volume measurements (Fig. 5A, top). In the BCM15100 model, the combination produced a greater reduction in tumor volume compared with either single agent, whereas in the TM00386 model, the combination did not significantly differ from PFK-158 monotherapy (Fig. 5A, top). Despite the differences in serial tumor volume measurements, endpoint tumor weights were significantly decreased in the combination-treated animals in both PDX models (Fig. 5A, bottom). To assess treatment-associated changes in proliferation, we performed immunohistochemical analysis of Ki67 (Fig. 5B; representative images shown for BCM15100). Combination-treated tumors exhibited significantly reduced Ki67 staining compared to control and palbociclib-treated tumors (Supplementary Fig. 3), consistent with decreased proliferative activity. PFKFB3 expression remained readily detectable across all treatment groups, consistent with the pharmacological mode of action of PFK-158. Notably, PFKFB3 staining suggested a trend toward increased expression in tumors treated with palbociclib alone and in combination, consistent with our in vitro findings demonstrating upregulation of PFKFB3 following CDK4/6 inhibition. Together, these findings indicate that pharmacological inhibition of PFKFB3 is associated with enhanced antitumor activity of CDK4/6 inhibition in vivo—despite preserved or potentially increased—PFKFB3 levels.

### CDK4/6 and PFKFB3 inhibition restricts glucose-derived carbon utilization *in vivo*

**Fig. 6:**
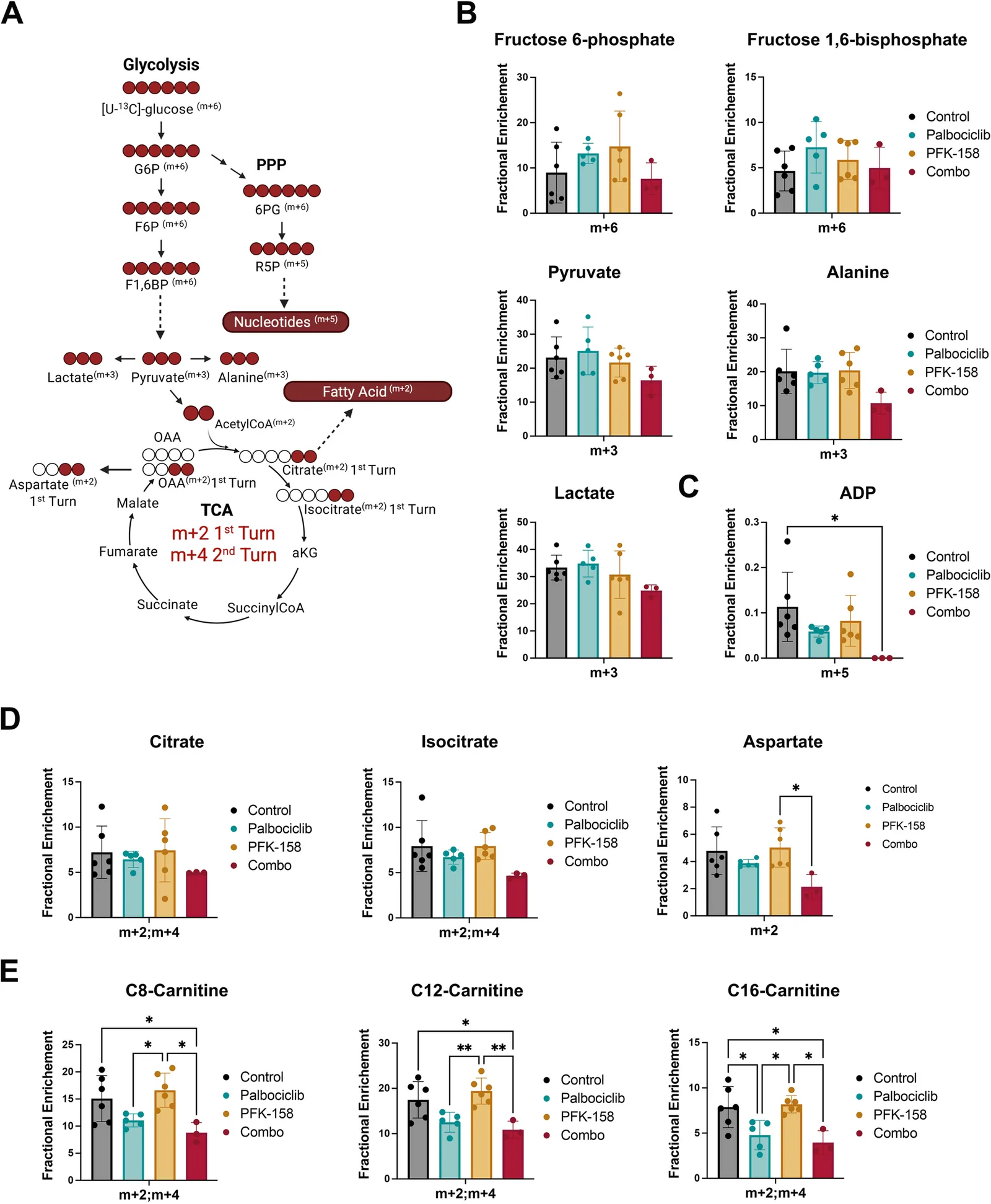
Metabolic analyses following combined CDK4/6 and PFKFB3 inhibition in vivo. TM00386 PDX-bearing mice were treated with vehicle control, 50 mg/kg palbociclib, 40 mg/kg PFK-158, or the combination, and subjected to in vivo [U-¹³C]-glucose bolus administration at endpoint, as described in Methods. **A** Schematic of [U-¹³C]-glucose tracing, illustrating carbon flow through glycolysis, diversion into the PPP, entry into the TCA cycle, and incorporation into amino acids, nucleotides, and fatty acid–associated metabolites. Expected isotopologues are indicated (m + 6 for hexose intermediates, m + 3 for pyruvate-derived metabolites, m + 5 for ribose-containing metabolites, and m + 2/m + 4 for metabolites derived from the first and second TCA cycle turns). **B** Fractional enrichment of glycolytic intermediates (fructose 6-phosphate, fructose 1,6-bisphosphate, pyruvate, alanine, and lactate). **C** Fractional enrichment (m + 5) of ADP. **D** Fractional enrichment of TCA cycle–related metabolites (citrate, isocitrate, and aspartate). **E** Fractional enrichment of acyl-carnitine species (C8-, C12-, and C16-carnitine; m + 2/m + 4). Data are presented as mean ± SD. Statistical analysis was performed using one-way ANOVA with Tukey’s post-hoc comparison

To determine how combined CDK4/6 and PFKFB3 inhibition alter glucose metabolism in vivo, we performed [U-^13^C]-glucose tracing at the experimental endpoint and quantified tumoral fractional enrichment across major metabolic pathways. The schematic in Fig. 6A summarizes the metabolic routes interrogated, illustrating glucose flux through glycolysis and its branching into the PPP, the tricarboxylic acid (TCA) cycle, and fatty acid biosynthesis.

We first assessed glucose-derived carbon incorporation into glycolytic intermediates as illustrated in Fig. 6A. Consistent with our in vitro findings, early glycolytic intermediates displayed detectable incorporation of ^13^C glucose-derived in all treatment groups (Fig. 6B). No statistically significant differences in fractional ¹³C enrichment of fructose 6-phosphate, fructose 1,6-bisphosphate, pyruvate, alanine, or lactate were observed between groups.

Next, we analyzed glucose entry into anabolic pathways, focusing on ribose labeling of ADP as a readout for PPP activity (Fig. 6C). Combination treatment significantly reduced m + 5 incorporation into ADP, whereas no statistically significant reduction was observed with palbociclib alone. These findings indicate that dual CDK4/6 and PFKFB3 inhibition suppresses nucleotide synthesis *in vivo.*

To determine whether glucose-derived carbon was incorporated into mitochondrial metabolism, we analyzed labeling of TCA cycle intermediates (Fig. 6D). Fractional enrichment of citrate and isocitrate (m + 2/m + 4) was not significantly different between groups. Aspartate m + 2 labeling, which reflects incorporation of glucose-derived carbon into oxaloacetate via PDH entry into the TCA cycle, showed a significant reduction in the combination treatment.

Lastly, we examined fatty acid labeling using short (C8)-, medium (C12)-, and long (C16)-chain acyl-carnitine labeling as a surrogate for incorporation of glucose-derived acetyl-CoA into fatty acids (Fig. 6E). Palbociclib significantly reduced the m + 2/m + 4 labeling of C8-, C12-, C16-carnitines with no additional statistically significant effect observed in the combination group. These findings indicate a marked block of glucose incorporation into fatty acids in vivo driven by palbociclib.

Taken together, these in vivo tracing studies demonstrate that dual CDK4/6 and PFKFB3 inhibition restricts the routing of glucose-derived carbons into nucleotide synthesis, with CDK4/6 inhibition alone significantly limiting incorporation into fatty acids and combination treatment further impacting select mitochondrial metabolites. This coordinated disruption of glucose-dependent anabolic and mitochondrial metabolism provides a mechanistic basis for the enhanced antitumor activity observed with dual CDK4/6 and PFKFB3 inhibition.

## Discussion

While cancer metabolism has long been considered a therapeutic vulnerability, the metabolic adaptations that emerge in response to targeted therapies, in particular CDK4/6 inhibitors, remain incompletely defined [29, 30]. Emerging evidence indicates that CDK4/6 signaling intersects with metabolic regulation through both RB-dependent transcriptional programs and direct effects on metabolic enzymes; however, how these changes reshape glucose utilization in tumors has not been fully elucidated [14, 31, 32]. Given the widespread adoption of CDK4/6 inhibitors as first-line therapy in combination with antiestrogens in ER+ breast cancer patients, a better understanding of their metabolic consequences is warranted.

In this study, we demonstrate that CDK4/6 inhibition induces a distinct metabolic state in ER+ breast cancer cells characterized by increased glycolytic flux through the PFKFB3-PFK1 regulatory step coupled and reduced routing of glucose-derived carbon into downstream biosynthetic pathways. Notably, this metabolic phenotype was observed across multiple clinically approved CDK4/6 inhibitors, despite their known differences in kinase selectivity and pharmacologic properties, indicating that rewiring of glycolytic regulation represents a conserved consequence of CDK4/6 pathway inhibition [33–35]. Palbociclib treatment increased glycolytic activity, as evidenced by accumulation F2,6BP, early glycolytic intermediates and enhanced labeling of fructose 1,6-bisphosphate. Notably, despite this increase in glycolytic flux, incorporation of glucose-derived carbons into nucleotide synthesis and fatty acid biosynthesis was markedly reduced. These findings indicate that CDK4/6 inhibition uncouples glycolytic flux from anabolic glucose utilization, consistent with reduced biosynthetic demand during G1 cell cycle arrest.

PFKFB3 has been extensively implicated in sustaining high glycolytic flux in breast cancer and other solid tumors, positioning it as an important regulatory node linking proliferative signaling to glucose metabolism [20, 22, 26, 36]. In our study, CDK4/6 inhibition consistently increased basal and compensatory glycolysis, and genetic reduction of PFKFB3 attenuated the CDK4/6 inhibitor-induced increase in glycolytic flux. Notably, the extent of this effect differed between cell lines, with a more pronounced dependence on PFKFB3 observed in MCF7 cells compared to T47D cells, where glycolytic activation was only partially attenuated. Despite this difference in glycolytic dependence, both cell lines exhibited comparable sensitivity to PFK-158 in vitro. This suggests that sensitivity to PFKFB3 inhibition is not solely determined by dependence on PFKFB3 for maintaining glycolysis and may reflect additional context-dependent factors, baseline expression levels or incomplete depletion of PFKFB3 following CRISPR. Additionally, PFKFB3 has been reported to exert functions beyond glycolytic regulation, including roles in cell cycle progression and autophagy [37–40], which may also contribute to the observed sensitivity of PFK-158 and warrants further investigation.

In contrast to our observations of increased glycolytic activity following CDK4/6 inhibition, Lorito et al., reported reduced glycolytic capacity upon CDK4/6 inhibition [19]. A likely explanation for this apparent contradiction lies in differences in normalization strategies. Normalization to total protein assumes constant protein content per cell, an assumption that may not hold when cell cycle arrest is accompanied by continued biomass accumulation, as occurs during CDK4/6 inhibitor–induced G1 arrest [23]. Under these conditions, protein-based normalization may underestimate glycolytic activity on a per-cell basis, whereas normalization to cell number provides a more direct assessment of metabolic activity per cell. Consistent with this, we observed that normalization to cell number revealed an increase in glycolytic flux that was attenuated when data were normalized to protein content. Together, these findings suggest that normalization strategy can influence the interpretation of metabolic responses to CDK4/6 inhibition. Together, these findings highlight the importance of normalization strategy when interpreting metabolic responses to CDK4/6 inhibition.

Our in vitro metabolomics data further support the increase in glycolysis in response to CDK4/6 inhibition as shown by the accumulation of glycolytic intermediates and increased carbon incorporation into fructose 1,6-bisphosphate. In vivo tracing studies revealed more modest changes in glycolytic intermediates, which is not unexpected given the complexity of the tumor microenvironment and the absence of fasting prior to tracer administration. Moreover, tumor bulk measurements incorporate stromal, immune and endothelial cells that raise the potential for diluting tumor-specific metabolic changes. Despite these confounding factors, directional increases in upstream glycolytic intermediates were preserved in vivo, supporting our conclusion that glycolysis is activated under CDK4/6 inhibition.

In contrast, the most pronounced metabolic effects observed in vivo occurred downstream of glycolysis, with reduced incorporation of glucose-derived carbon into lipid-associated metabolites and nucleotides. These changes are consistent with reduced demand for *de novo* lipid synthesis and nucleotide production during CDK4/6-induced cell cycle arrest. Proliferating tumor cells require sustained anabolic flux to support membrane biogenesis and DNA replication; thus, inhibition of proliferation would be expected to limit glucose routing into lipogenic and nucleotide biosynthetic pathways. Consistent with this framework, both in vitro and in vivo metabolomic analyses demonstrated reduced glucose contribution to nucleotide pools. Prior studies have shown that palbociclib suppresses PPP activity through inhibition of glucose 6-phosphate dehydrogenase, resulting in diminished ribose 5-phosphate production and impaired nucleotide synthesis [16]. Although these studies were performed in a different tumor type, they provide independent support for the finding that CDK4/6 inhibition limits glucose entry into nucleotide biosynthesis. Together, these findings support a model in which reduced nucleotide labeling reflects decreased anabolic demand reinforced by restricted pentose phosphate pathway flux, rather than impaired glucose uptake or glycolytic capacity. A limitation of this study is that bulk metabolomics analysis does not resolve cell type-specific metabolic responses within the tumor microenvironment; future studies incorporating spatial or single cell approaches will be valuable to further refine these findings.

Importantly, despite reduced downstream anabolic glucose utilization, glycolytic activity itself remains elevated under CDK4/6 inhibition and likely serves functions beyond biomass production, including maintenance of ATP levels, redox balance, and metabolic homeostasis in G1-arrested cells. In this context, glycolysis appears to support tumor cell maintenance rather than proliferation. Disruption of this adaptive metabolic state through PFKFB3 inhibition limits glucose utilization and may compromise the ability of CDK4/6-inhibited cells to maintain metabolic balance.

Consistent with this framework, co-targeting PFKFB3 further restricted glucose utilization across multiple downstream pathways and enhanced the antitumor efficacy of CDK4/6 inhibition in vivo. Together, these findings support a model in which CDK4/6 inhibition rewires metabolism to reduce biosynthetic demand while simultaneously increasing reliance on regulated glycolytic flux for metabolic homeostasis. Targeting PFKFB3 exploits this adaptive metabolic vulnerability and provides a mechanistic rationale for combining glycolytic inhibition with CDK4/6-targeted therapy in ER+ breast cancer. Future studies comparing PFKFB3 inhibition with broader glycolytic inhibitors will be important to define whether the therapeutic effects observed here reflect general glycolytic suppression or are specific to PFKFB3 targeting.

## Conclusions

Our findings demonstrate that CDK4/6 inhibition rewires glucose metabolism in ER⁺ breast cancer by increasing glycolytic flux while limiting downstream anabolic utilization. Targeting this adaptive metabolic state through PFKFB3 inhibition enhances the antitumor efficacy of CDK4/6 inhibitors and supports the therapeutic potential of combining glycolytic modulation with CDK4/6-directed therapy.

## Supplementary Information

Supplementary Material 1.

## Data Availability

No datasets were generated or analysed during the current study.

## Abbreviations

ADP: Adenosine diphosphate
AI: Aromatase inhibitor
ANOVA: Analysis of variance
AMP: Adenosine monophosphate
ATP: Adenosine triphosphate
Cas9: CRISPR–associated protein 9
CDK4/6: Cyclin–dependent kinase 4 and 6
CRISPR: Clustered regularly interspaced short palindromic repeats
ECAR: Extracellular acidification rate
ER / ER⁺: Estrogen receptor / estrogen receptor–positive
ERα: Estrogen receptor alpha
F2,6BP: Fructose–2,6–bisphosphate
F6P: Fructose–6–phosphate
G1: Gap 1 phase of the cell cycle
G6P: Glucose–6–phosphate
GLUT1: Glucose transporter 1
HER2: Human epidermal growth factor receptor 2
HK2: Hexokinase II
LC/MS: Liquid chromatography–mass spectrometry
LDHA: Lactate dehydrogenase A
OAA: Oxaloacetate
PDH: Pyruvate dehydrogenase
PDX: Patient–derived xenograft
PFK: 1–Phosphofructokinase–1
PFKFB3: 6–Phosphofructo–2–kinase/fructose–2,6–bisphosphatase 3
pPFKFB3 (Ser461): Phosphorylated PFKFB3 at serine 461
PPP: Pentose phosphate pathway
R5P: Ribose–5–phosphate
Rb: Retinoblastoma protein
Ribo: Ribociclib
SD: Standard deviation
TCA: Tricarboxylic acid (cycle)
¹³C: Carbon–13 isotope
