# Supplementary material for: Targeting PFKFB3 to enhance CDK4/6 inhibitor response in ER+ breast cancer
Source: Breast Cancer Res. 2026 Jun 25;28:153. doi: 10.1186/s13058-026-02336-2 (PMC13556003; doi:10.1186/s13058-026-02336-2)
Supplement: Supplementary file 1 [file 13058_2026_2336_MOESM1_ESM.zip › Supplemental Figure 3 Legend.docx]

**Supplemental Figure 3. Quantification of Ki67-positive cells in BCM15100 tumors.** Ki67 immunohistochemical staining was quantified using QuPath v0.6.0. For each tumor, five independent regions of interest (ROIs) within viable tumor areas were analyzed, and the percentage of Ki67-positive nuclei was determined. ROI values were averaged to generate a single value per tumor. Data are presented as mean ± SEM. Statistical significance was determined by one-way ANOVA with Tukey’s post-hoc comparison.
