# Supplementary material for: Targeting PFKFB3 to enhance CDK4/6 inhibitor response in ER+ breast cancer
Source: Breast Cancer Res. 2026 Jun 25;28:153. doi: 10.1186/s13058-026-02336-2 (PMC13556003; doi:10.1186/s13058-026-02336-2)
Supplement: Supplementary file 1 [file 13058_2026_2336_MOESM1_ESM.zip › Supplemental Figure legends FN 040626.docx]

Supplemental Figure legends

**Supplemental Figure 1. Densitometric quantification of glycolytic enzyme expression following CDK4/6 inhibition.** Densitometric analysis of GLUT1, HKII, PFK1, PKM2, PDH, LDHA, and MCT4 protein levels in MCF7 and T47D cells treated with palbociclib (PD) or ribociclib (Ribo) for 24 or 48 hours. Protein levels were normalized to β-actin and expressed relative to control. Data are shown as individual values with SD indicated from three biological replicates. Statistical analysis was performed using one-way ANOVA with multiple comparisons; no statistically significant changes were observed across conditions unless indicated.

**Supplemental Figure 2. 2PG/3PG and pyruvate abundance and fractional enrichment following CDK4/6 inhibition and [U-¹³C]-glucose tracing.** Relative abundance (top) and fractional enrichment (bottom) of glycolytic intermediates 2-phosphoglycerate/3-phosphoglycerate (2PG/3PG) and pyruvate following [U-¹³C]-glucose tracing in MCF7 cells treated with control or 0.5 µM palbociclib. Fractional enrichment of the m+3 isotopologue is shown for each metabolite. Data are presented as individual values ± SD from one experiment with 5 technical replicates. Paired two-tailed Student’s t-tests were used for statistical analysis (***P < 0.001).
