# Supplementary material for: Targeting PFKFB3 to enhance CDK4/6 inhibitor response in ER+ breast cancer
Source: Breast Cancer Res. 2026 Jun 25;28:153. doi: 10.1186/s13058-026-02336-2 (PMC13556003; doi:10.1186/s13058-026-02336-2)

Western Blots Figure 1:

MCF7 Cells:
24hr blots

PhosphoFB3:
F02_23_MCF7pS_WCE_24_PhosphoPFKFB3_001.tif

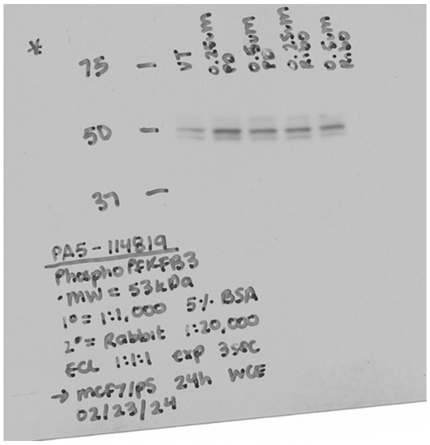

PFKFB3:
F02_23_MCF7pS_WCE_24_PFKFB3_001

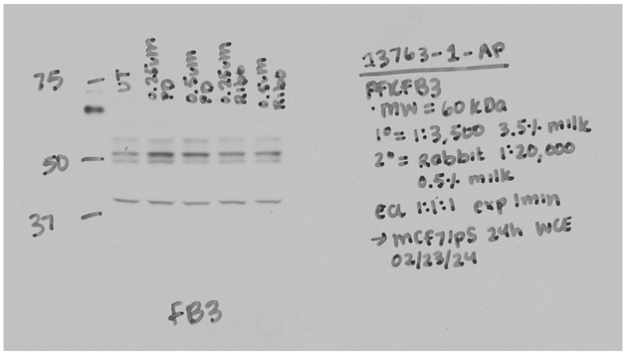

GLUT1:
W02_28_MCF7pS_WCE_24_Glut1_001.tif

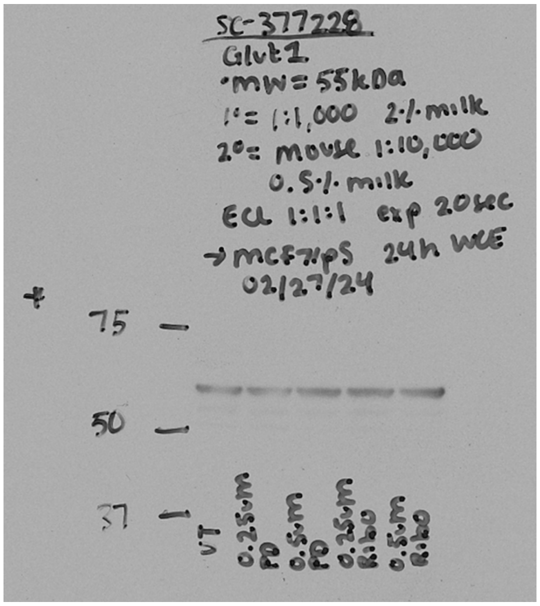

HKII:
W02_28_MCF7pS_WCE_24_HK2_001.tif

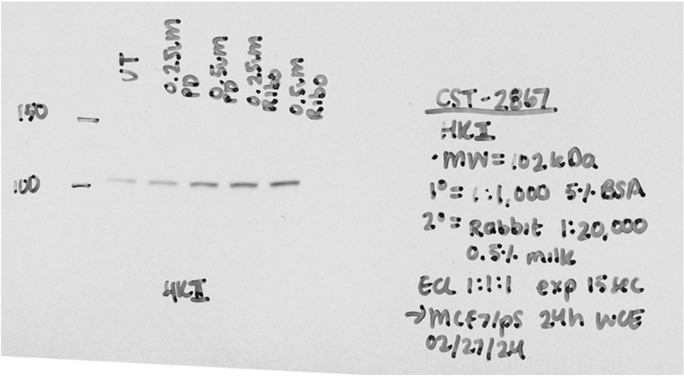

PFK1:
F02_23_MCF7pS_WCE_24_PFK_002.tif

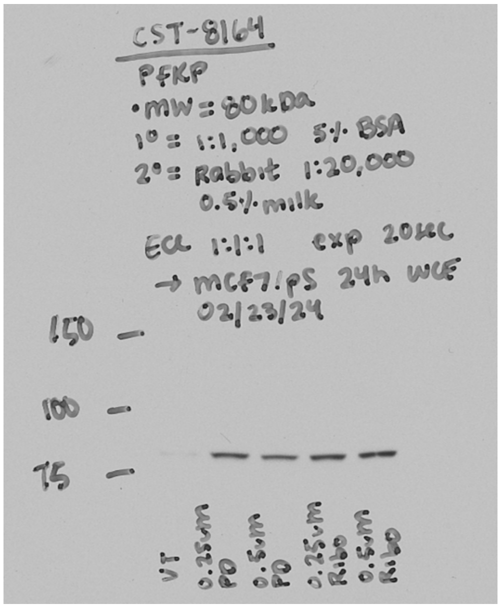

PDH:
W02_28_MCF7pS_WCE_24_PDH_001.tif

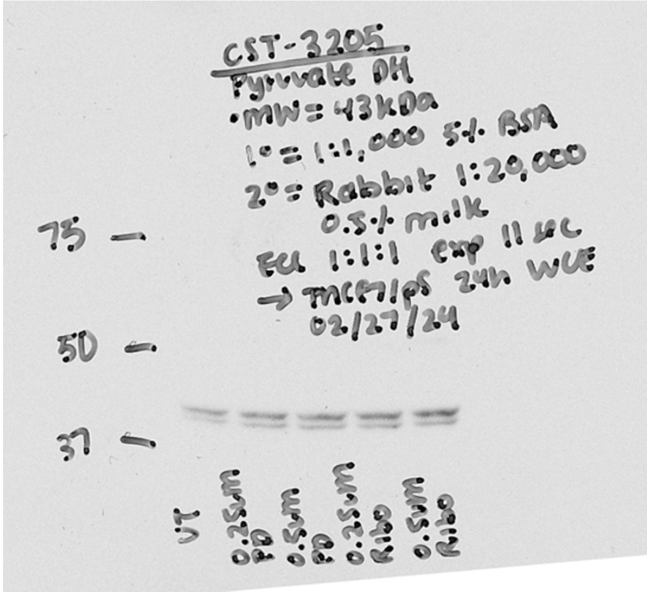

MCT4:
W02_28_MCF7pS_WCE_24_MCT4_001.tif

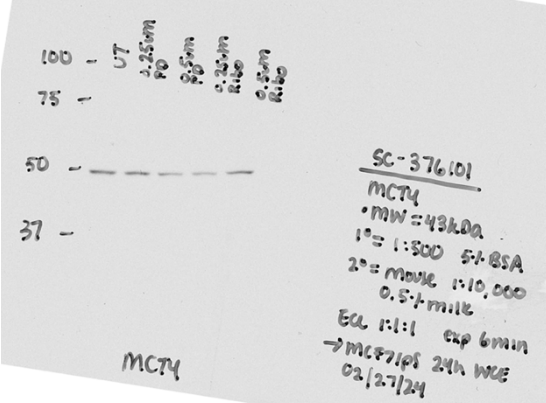

Actin:
F03_01_MCF7pS_WCE_24_Actin_002.tif

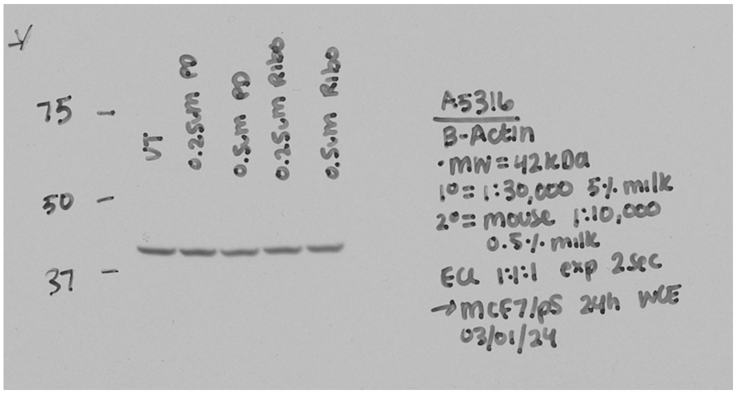

LDHA:
F02_23_MCF7pS_WCE_24_LDHA_001.tif

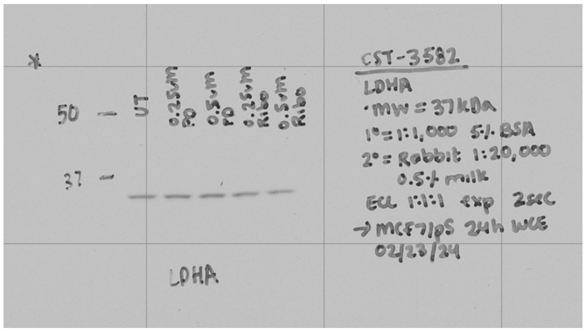

48hr blots

PhosphoFB3:
Th01_04_MCF7pS_WCE_48_
PhosphoPFKFB3_002.tif

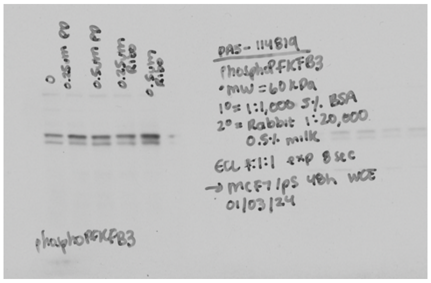

PhosphoFB3:
Th01_04_MCF7pS_WCE_48h
_PFKFB3_001.tif

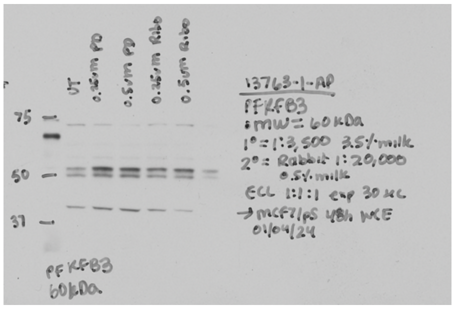

GLUT1:
W01_10_MCF7pS_WCE_48h
_Glut1_001.tif

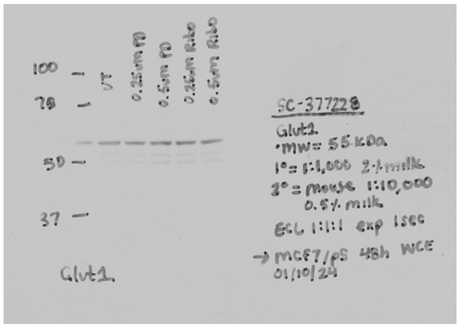

HK2:
Th01_04_MCF7pS_WCE_48h
_HK2_002.tif

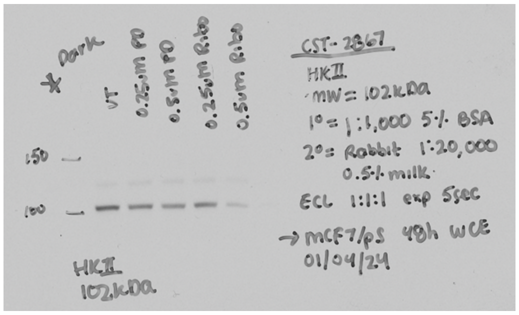

PFK1:
F01_05_MCF7pS_WCE_48h_
PFKP_001.tif

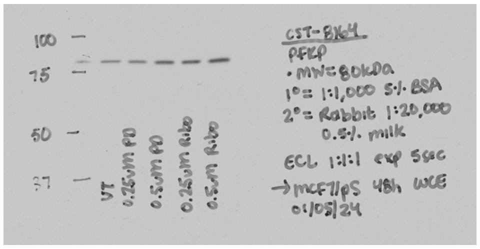

PDH:
Th01_04_MCF7pS_WCE_48h_
PDH_002.tif

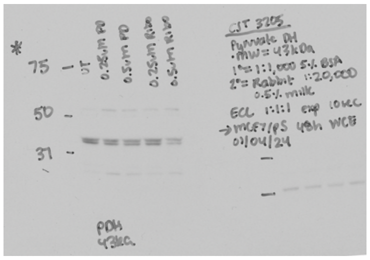

LDHA:
Th01_04_MCF7pS_WCE_48h_
LDHA_003.tif

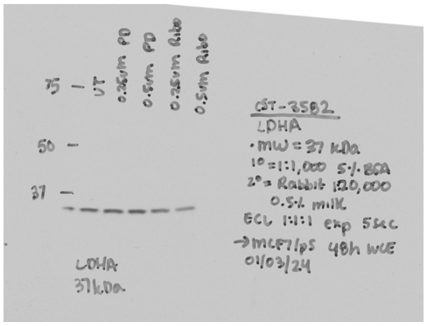

MCT4:
F01_05_MCF7pS_WCE_48h_
MCT4_001.tif

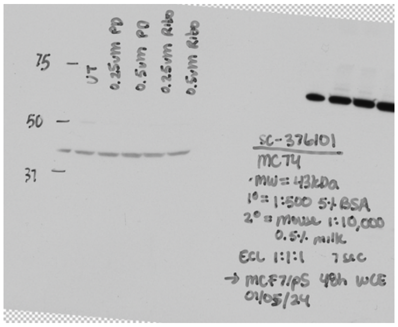

Actin: Th01_04_MCF7pS_WCE_48h_Actin_001.tif

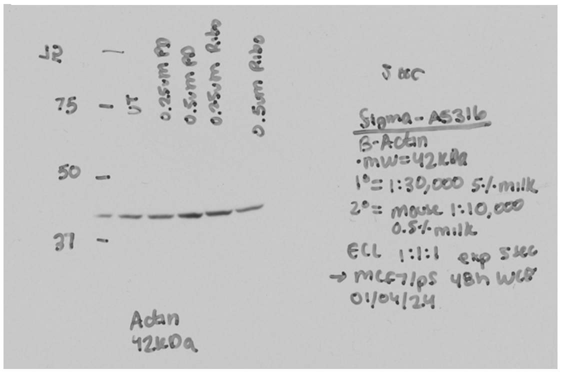

Supplement: Supplementary file 1 [file 13058_2026_2336_MOESM1_ESM.zip › Western Blots Figure 1 original scans MCF7 CELLS.pdf]
