# Supplementary material for: Targeting PFKFB3 to enhance CDK4/6 inhibitor response in ER+ breast cancer
Source: Breast Cancer Res. 2026 Jun 25;28:153. doi: 10.1186/s13058-026-02336-2 (PMC13556003; doi:10.1186/s13058-026-02336-2)
Supplement: Supplementary file 1 [file 13058_2026_2336_MOESM1_ESM.zip › Western Blots Figure 1 original scans T47D CELLS.pdf]

Western Blots Figure 1:

T47D Cells:
24hr blots

PhosphoFB3:
M03_18_T47DpS_WCE_24_PhosphoPFKFB3_001.tif

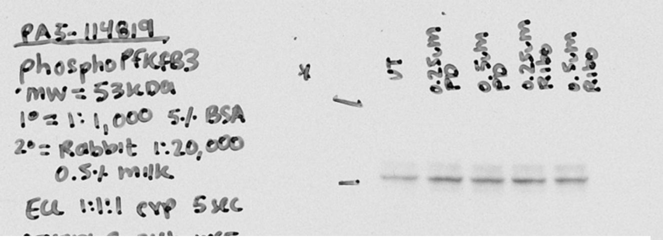

PFKFB3:
M03_18_T47DpS_WCE_24_PFKFB3_003.tif

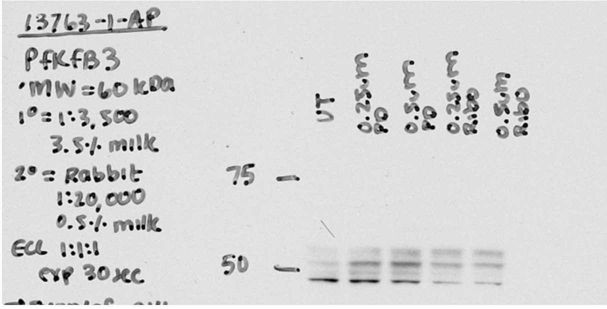

GLUT1:
M03_18_T47DpS_WCE_24_Glut1_002.tif

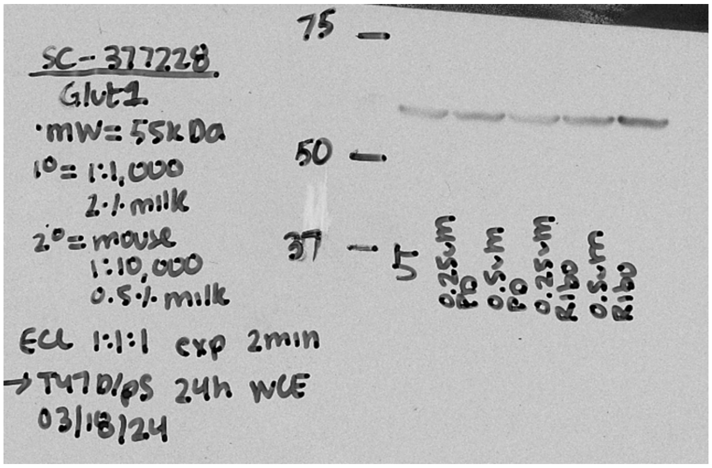

HKII:
M03_18_T47DpS_WCE_24_HK2_002.tif

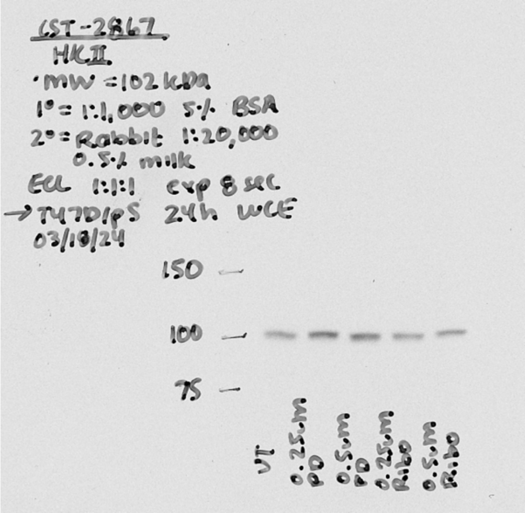

LDHA:
M03_18_T47DpS_WCE_24_LDHA_003.tif

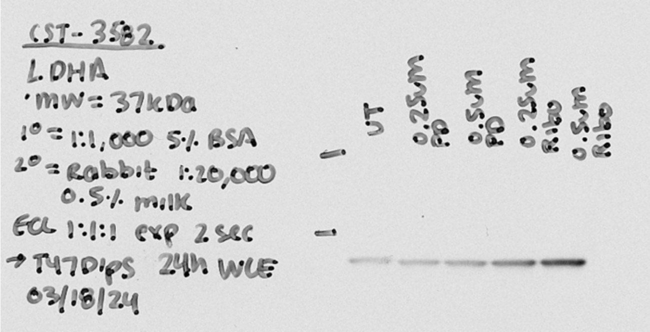

PFK1:
M03_18_T47DpS_WCE_24_PFKP_001.tif

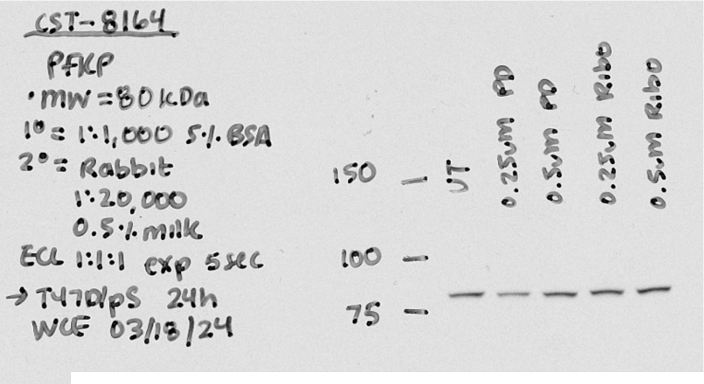

MCT4:
M03_18_T47DpS_WCE_24_MCT4_001.tif

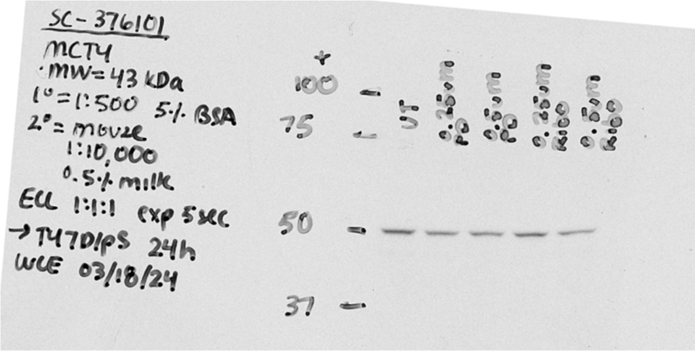

Actin:
M03_18_T47DpS_WCE_24_Actin_001.tif

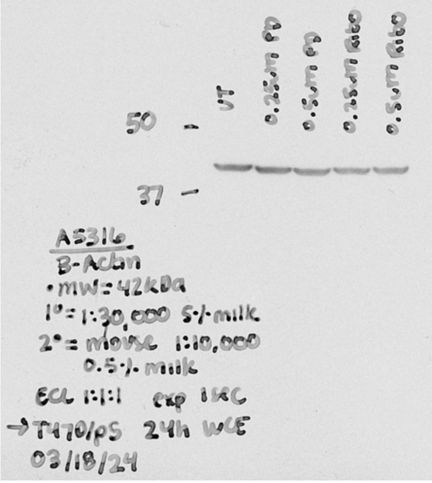

PDH:
M03_18_T47DpS_WCE_24_PDH_001.tif

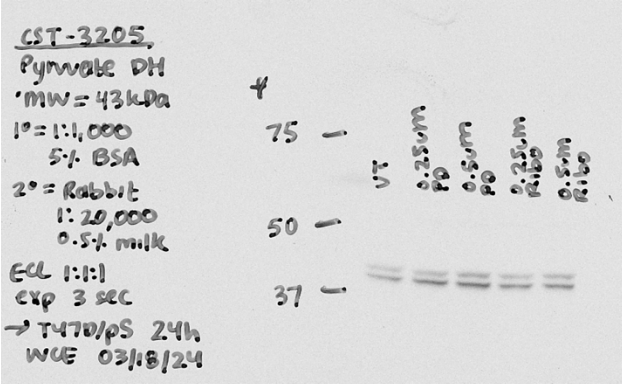

48hr blots

M03_18_T47DpS_WCE_48_PhosphoPFKFB3_004.tif

M03_18_T47DpS_WCE_48_PFKFB3_002.tif

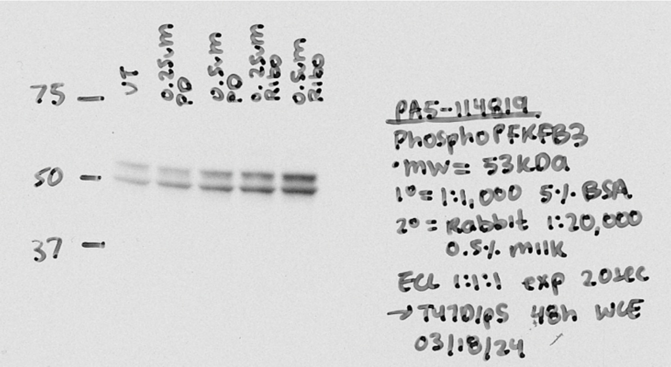

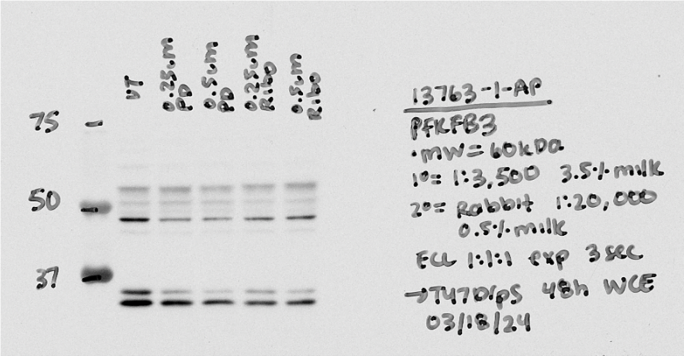

M03_18_T47DpS_WCE_48_Glut1_002.tif

M03_18_T47DpS_WCE_48_HK2_001.tif

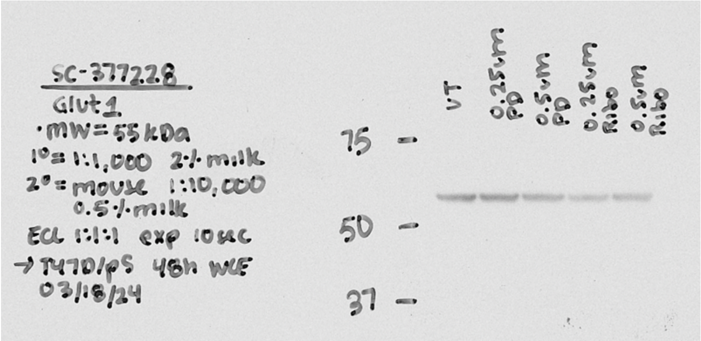

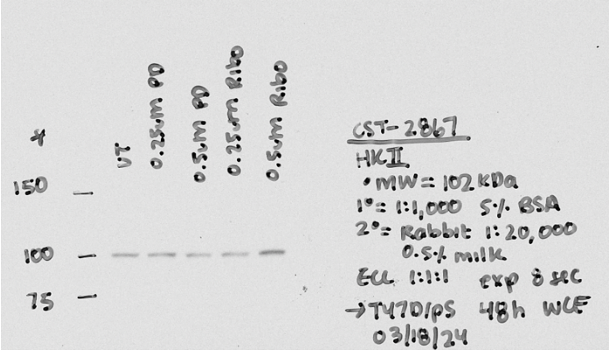

M03_18_T47DpS_WCE_48_PFKP_002.tif

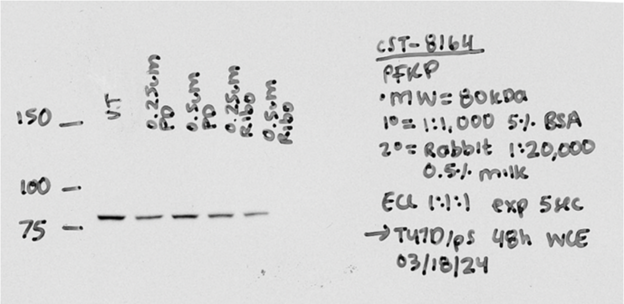

M03_18_T47DpS_WCE_48_PDH_002.tif

M03_18_T47DpS_WCE_48_LDHA_002.tif

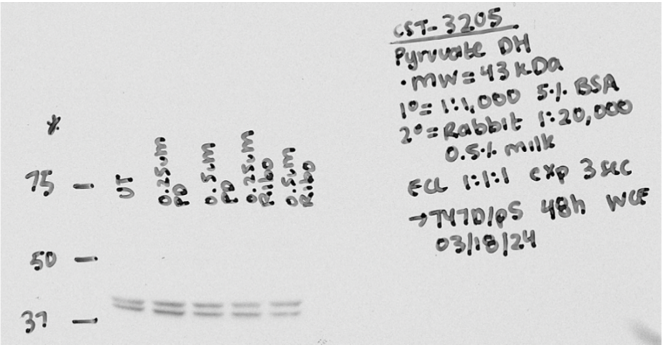

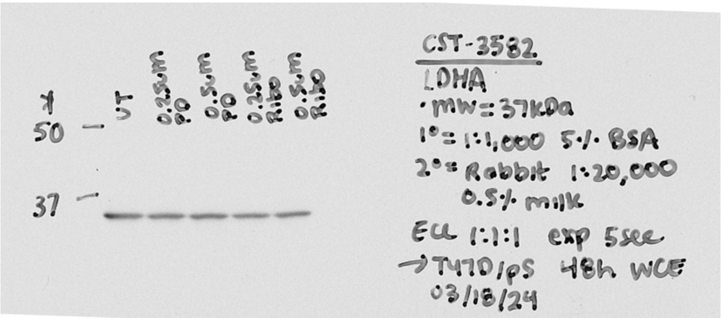

M03_18_T47DpS_WCE_48_MCT4_003.tif

M03_18_T47DpS_WCE_48_Actin_002.tif

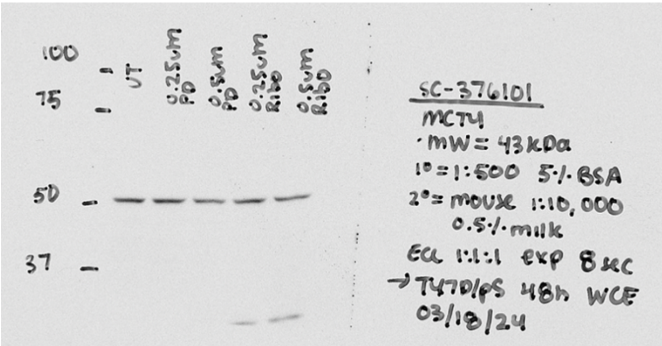
100
75
50
37
UT
0.25uM PD
0.5uM PD
0.25uM Ribo
0.5uM Ribo
SC-376101
MCT4
• MW = 43kDa
1° = 1:500 5% BSA
2° = mouse 1:10,000
0.5% milk
ECL 1:1:1 exp 8sec
→ T47D/PS 48h WCE
03/18/24

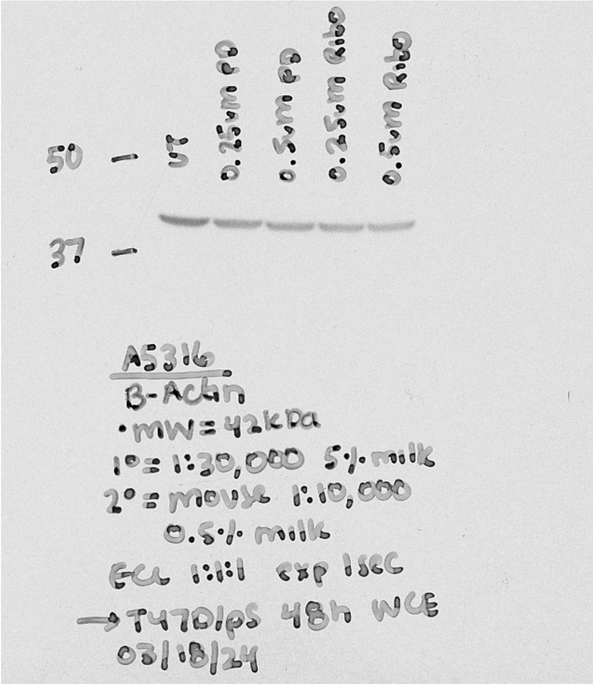
50
37
UT
0.25uM PD
0.5uM PD
0.25uM Ribo
0.5uM Ribo
A5316
B-Actin
• MW = 42kDa
1° = 1:30,000 5% milk
2° = mouse 1:10,000
0.5% milk
ECL 1:1:1 exp 1sec
→ T47D/PS 48h WCE
03/18/24
