# Supplementary material for: Targeting PFKFB3 to enhance CDK4/6 inhibitor response in ER+ breast cancer
Source: Breast Cancer Res. 2026 Jun 25;28:153. doi: 10.1186/s13058-026-02336-2 (PMC13556003; doi:10.1186/s13058-026-02336-2)

Western Blots **2026 REVISION** Figure 1:

MCF7 Cells:
24hr blots

PhosphoFB3: pPFKFB3
MCF7pS_PD.Ribo24h_WCE_04.1.26.tif

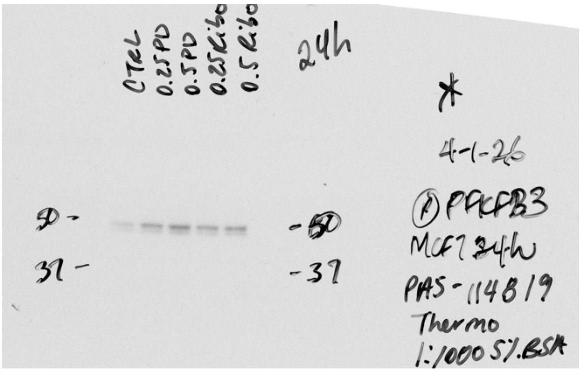

PFKFB3: PFKFB3
MCF7pS_PD.Ribo24h_WCE_04.1.26.tif

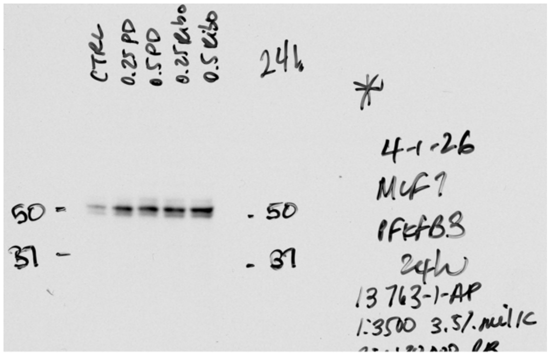

GLUT1: Glut1
MCF7pS_PD.Ribo24h_WCE_03.31.26dark
.tif

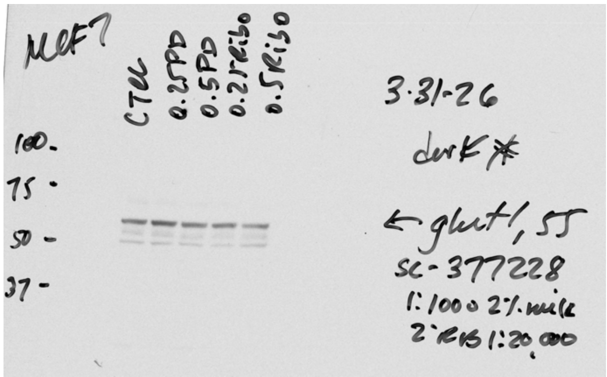

HKII: HKII
MCF7pS_PD.Ribo24h_WCE_03.31.26dark
.tif

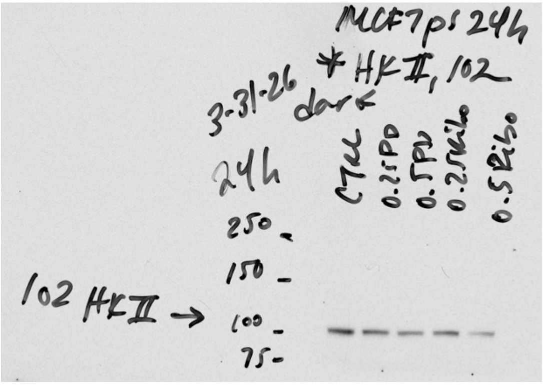

PFK1: PFK1
MCF7pS_PD.Ribo24h_WCE_03.31.26.tif

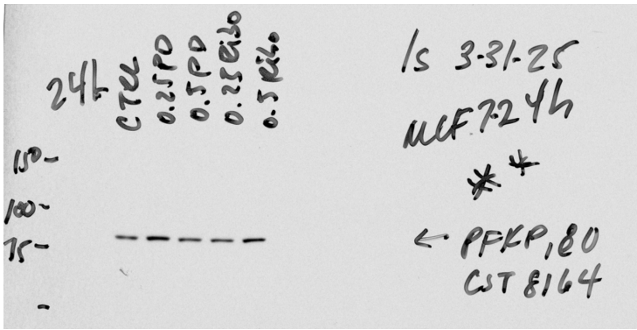

PKM2: PKM2
MCF7pS_PD.Ribo24h_WCE_03.31.26.tif

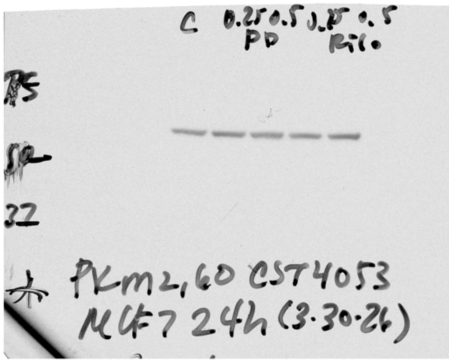

PDH: PDH
MCF7pS_PD.Ribo24h_WCE_04.1.26.tif

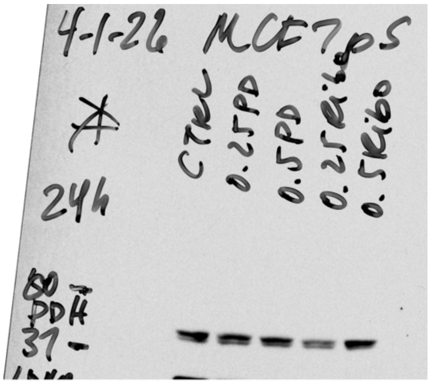

LDHA: LDHA
MCF7pS_PD.Ribo24h_WCE_03.31.26.tif

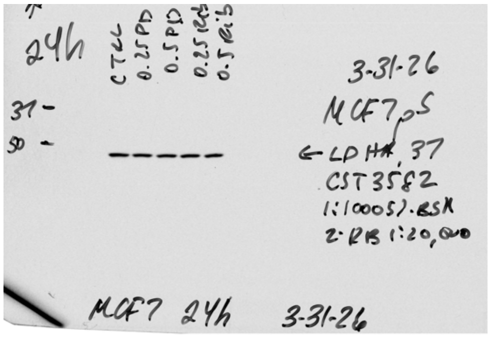

MCT4: MCT4
MCF7pS_PD.Ribo24h_WCE_04.3.26.tif

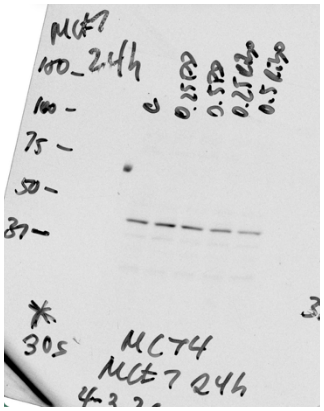

Actin: bActin
MCF7pS_PD.Ribo24h_WCE_04.2.26.tif

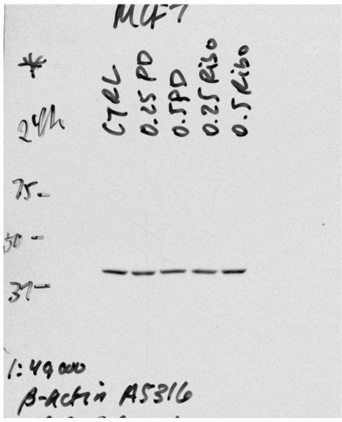

48hr blots

PhosphoFB3: pPFKFB3
MCF7pS_PD.Ribo48h_WCE_04.1.26.tif

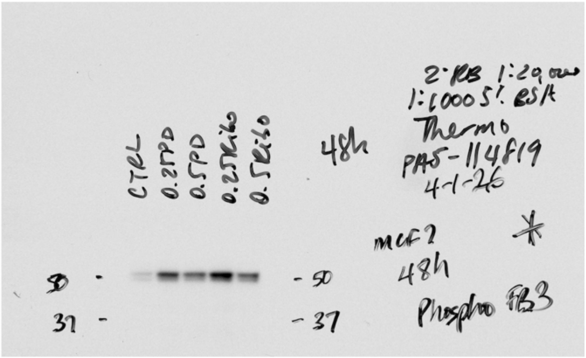

PhosphoFB3: PFKFB3
MCF7pS_PD.Ribo48h_WCE_04.1.26.tif

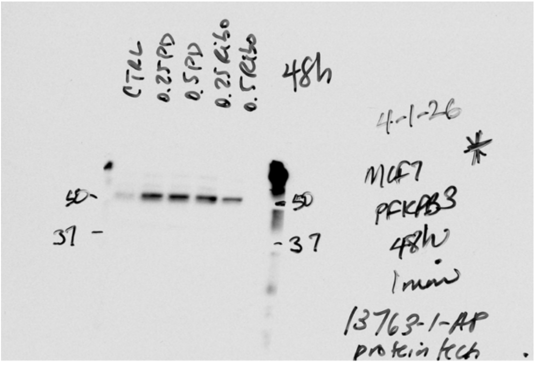

GLUT1: Glut1
MCF7pS_PD.Ribo48h_WCE_03.31.26.tif

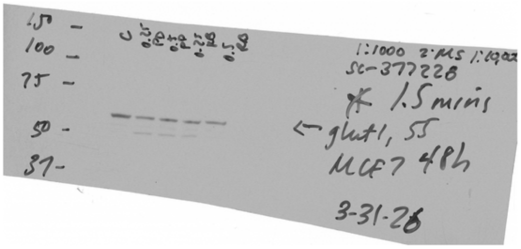

HII: HKII
MCF7pS_PD.Ribo48h_WCE_04.1.26.tif

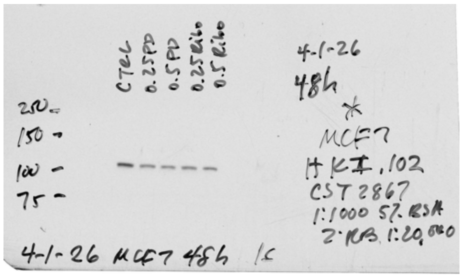

PFK1: PFKP
MCF7pS_PD.Ribo48h_WCE_03.31.26.tif

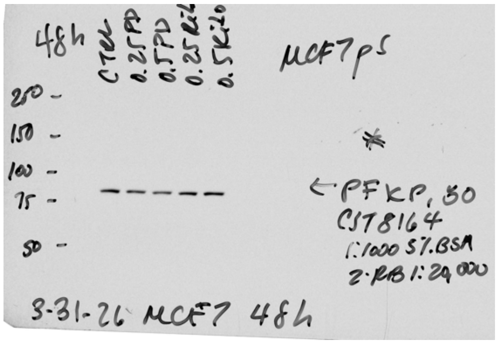

PKM2: PKM2
MCF7pS_PD.Ribo48h_WCE_04.03.26.tif

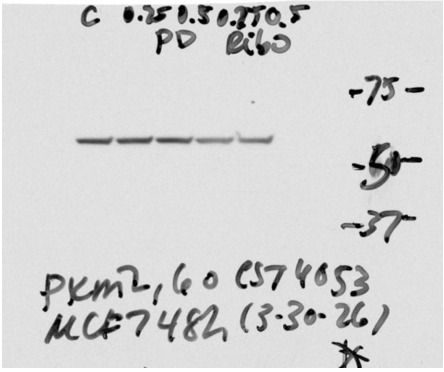

PDH: PDH
MCF7pS_PD.Ribo48h_WCE_03.31.26.tif

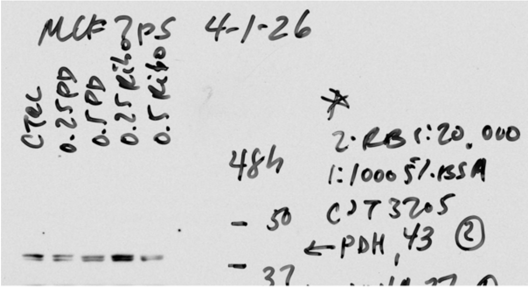

LDHA: LDHA
MCF7pS_PD.Ribo48h_WCE_03.31.26.tif

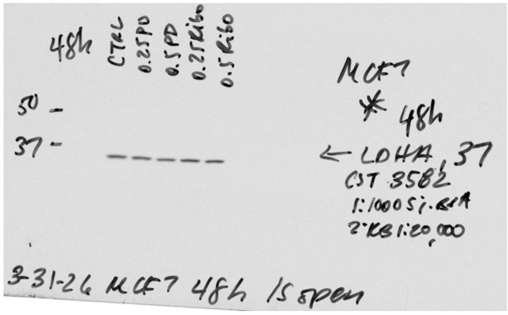

MCT4: MCT4
MCF7pS_PD.Ribo48h_WCE_04.3.26.tif

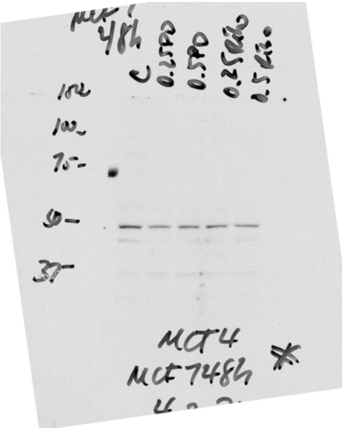

Actin: bActin
MCF7pS_PD.Ribo48h_WCE_04.2.26.tif

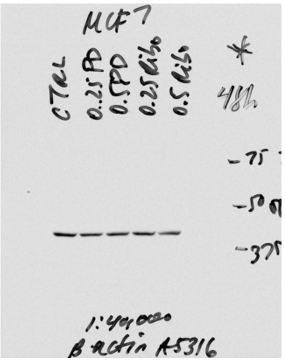

Supplement: Supplementary file 1 [file 13058_2026_2336_MOESM1_ESM.zip › Western Blots Figure 1 REVISION scans 24-48hr MCF7 CELLS.pdf]
