# Supplementary material for: Targeting PFKFB3 to enhance CDK4/6 inhibitor response in ER+ breast cancer
Source: Breast Cancer Res. 2026 Jun 25;28:153. doi: 10.1186/s13058-026-02336-2 (PMC13556003; doi:10.1186/s13058-026-02336-2)

Western Blots **2026 REVISION** Figure 1:

T47D Cells:

24hr blots

PhosphoFB3: pPFKFB3
T47DpS_PD.Ribo24h_WCE_03.27.26.tif

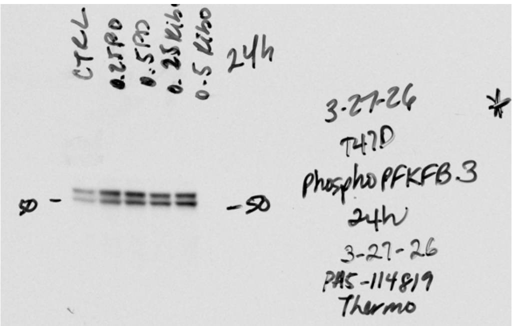

PFKFB3: PFKFB3
T47DpS_PD.Ribo24h_WCE_03.27.26.tif

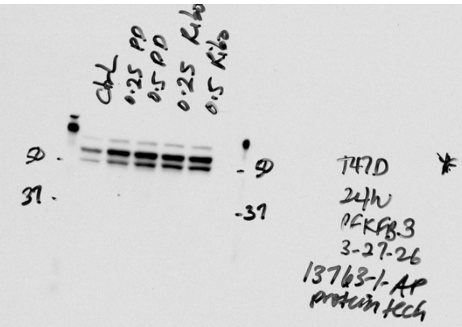

GLUT1: Glut1
T47DpS_PD.Ribo24h_WCE_03.26.26.tif

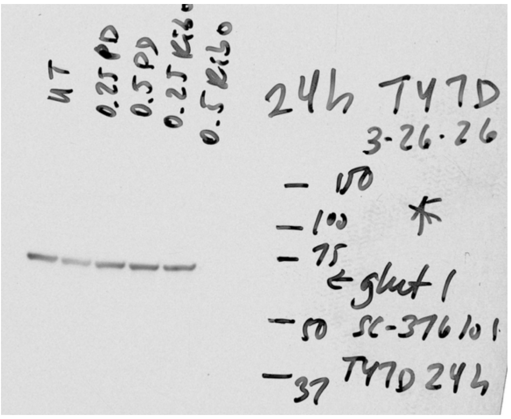

HKII: HKII
T47DpS_PD.Ribo24h_WCE_03.26.26light.

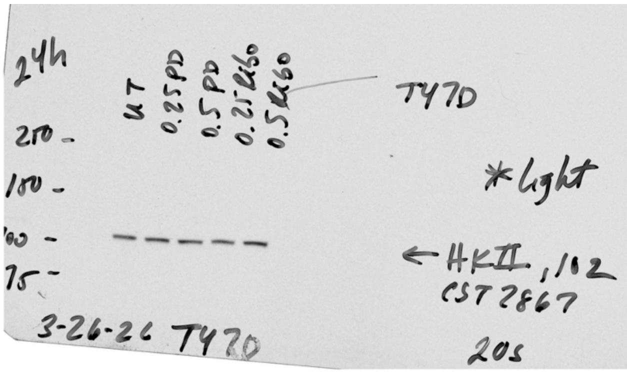

PFK1: PFKP
T47DpS_PD.Ribo24h_WCE_03.26.26.tif

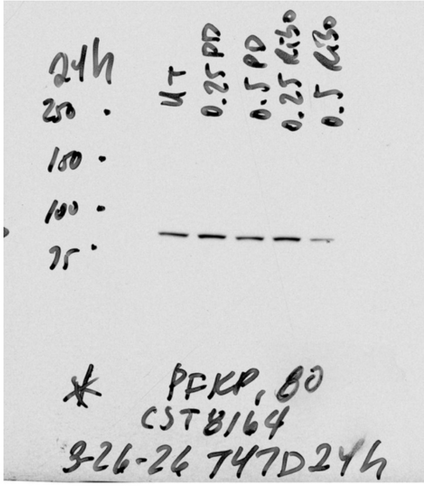

PKM2: PKM2
T47DpS_PD.Ribo24h_WCE_04.02.26.tif

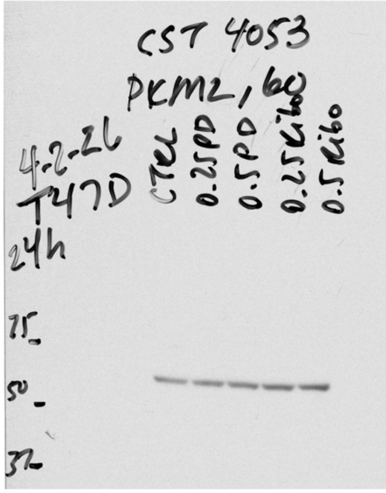

PDH: PDH
T47DpS_PD.Ribo24h_WCE_03.27.26.tif

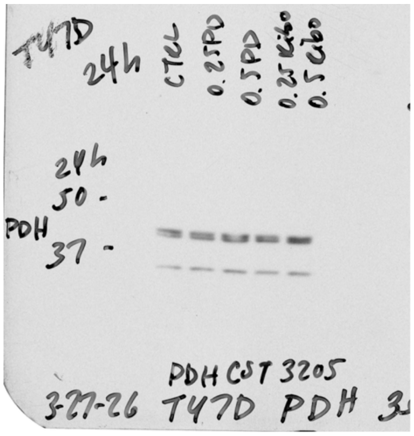

LDHA: LDHA
T47DpS_PD.Ribo24h_WCE_03.26.26.tif

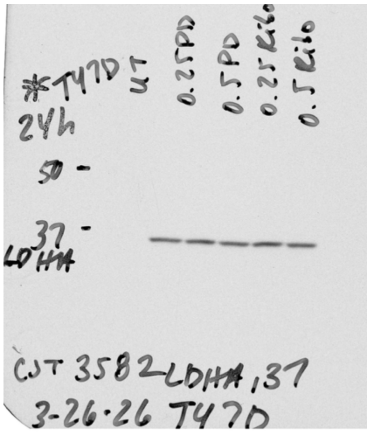

MCT4: MCT4
T47DpS_PD.Ribo24h_WCE_03.27.26.tif

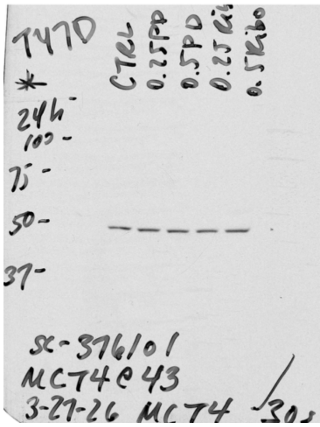

Actin: bActin
T47DpS_PD.Ribo24h_WCE_03.27.26.tif

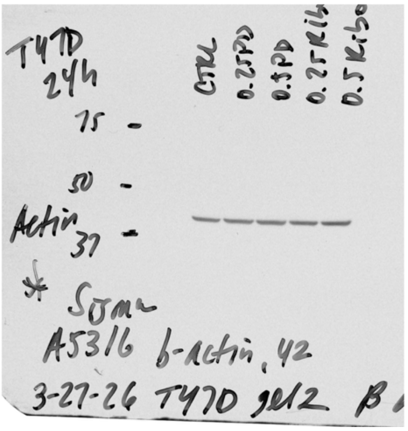

48hr blots

PhosphoFB3: pPFKFB3
T47DpS_PD.Ribo48h_WCE_03.27.26.tif

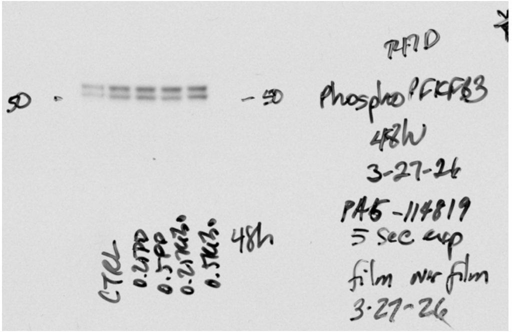

PFKFB3: PFKFB3
T47DpS_PD.Ribo48h_WCE_03.27.26.tif

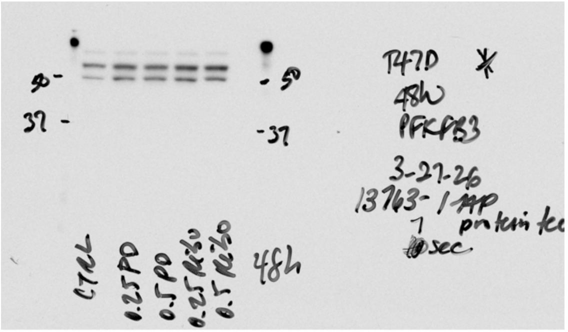

GLUT1: Glut1
T47DpS_PD.Ribo48h_WCE_03.26.26dark.tif

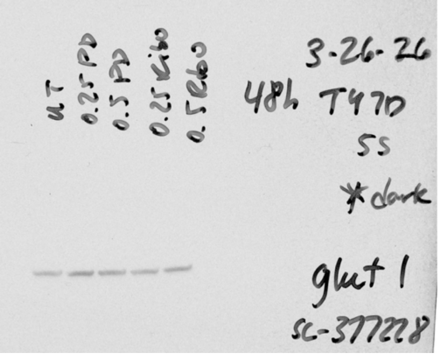

HKII: HKII
T47DpS_PD.Ribo48h_WCE_03.26.26.tif

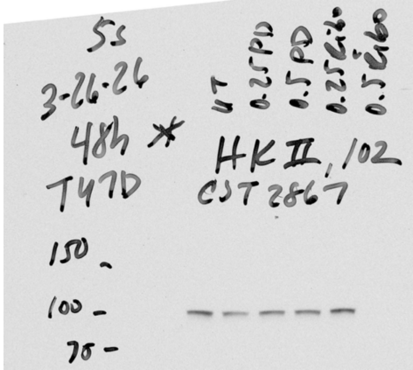

PFK1: PFKP
T47DpS_PD.Ribo48h_WCE_03.26.26.tif

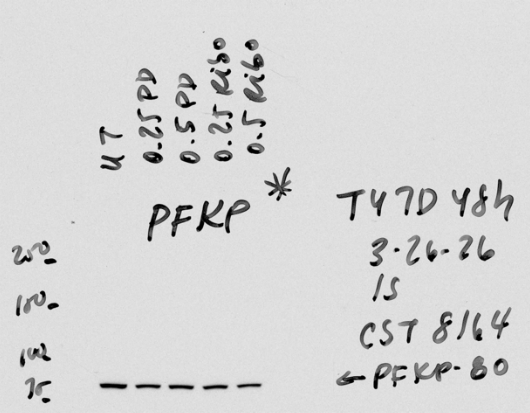

PKM2: PKM2
T47DpS_PD.Ribo48h_WCE_04.02.26.tif

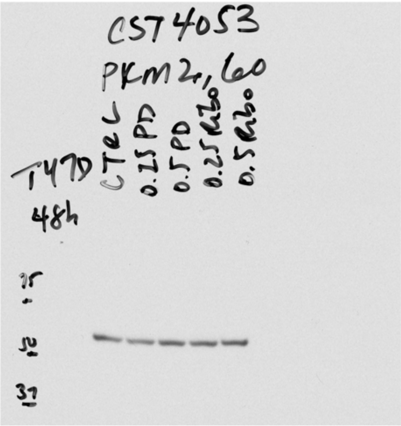

PDH: PDH
T47DpS_PD.Ribo48h_WCE_03.27.26.tif

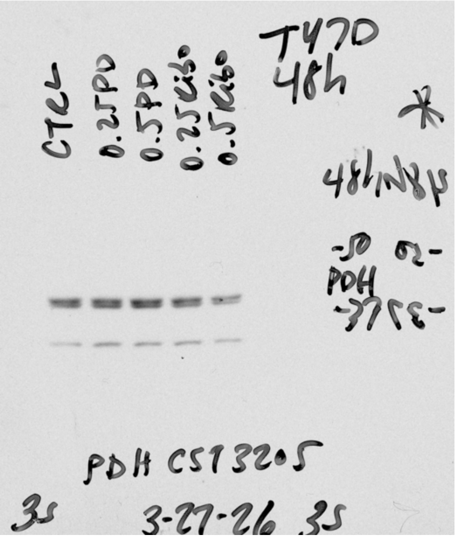

LDHA: LDHA
T47DpS_PD.Ribo48h_WCE_03.26.26.tif

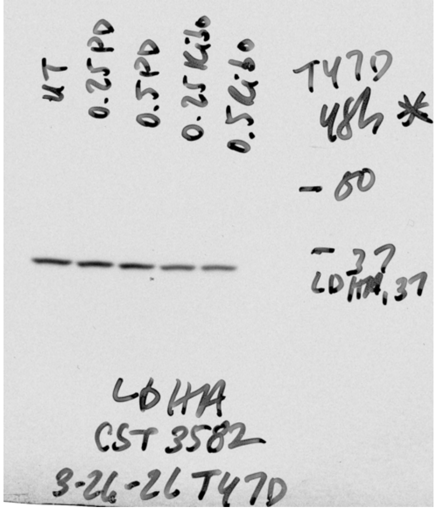

MCT4: MCT4
T47DpS_PD.Ribo48h_WCE_03.27.26.tif

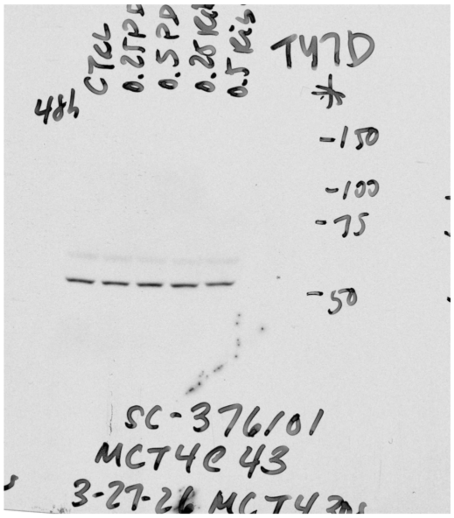

Actin: bActin
T47DpS_PD.Ribo48h_WCE_03.27.26light.tif

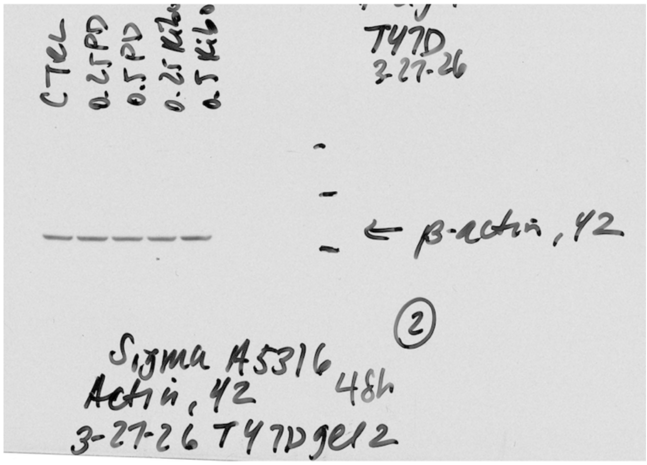

Supplement: Supplementary file 1 [file 13058_2026_2336_MOESM1_ESM.zip › Western Blots Figure 1 REVISION scans 24-48hrs T47D CELLS.pdf]
