# Supplementary material for: Targeting PFKFB3 to enhance CDK4/6 inhibitor response in ER+ breast cancer
Source: Breast Cancer Res. 2026 Jun 25;28:153. doi: 10.1186/s13058-026-02336-2 (PMC13556003; doi:10.1186/s13058-026-02336-2)

Western Blots Figure 3 (FN FIGURE):

FB3 CRISPR cells treated with 0.5 μM PD or Ribo for 48hrs.
Gel and WB by SMD 07-29-25. Gel included control cells that we did not include in the manuscript. Fn figure has only lanes 4-12

MCF7 Cells

PFKFB3 blot: FB3 MCF7pS PFKFB3CRISPR350-355 PD-RB Run1 7-31-25.tif

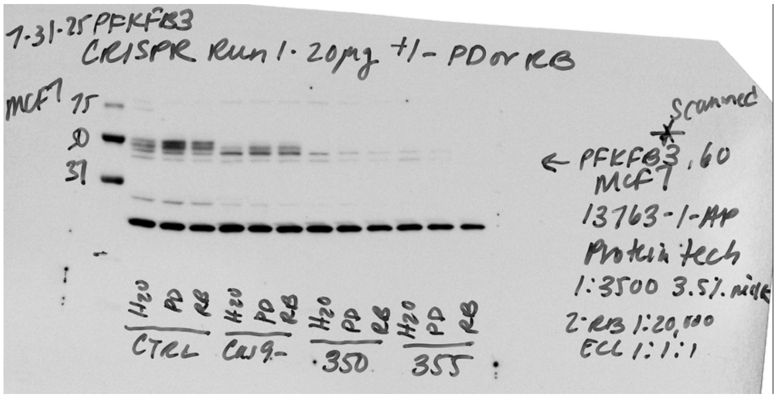

Actin blot: bActin MCF7pS PFKFB3CRISPR350-355 PD-RB Run1 8-5-25.tif

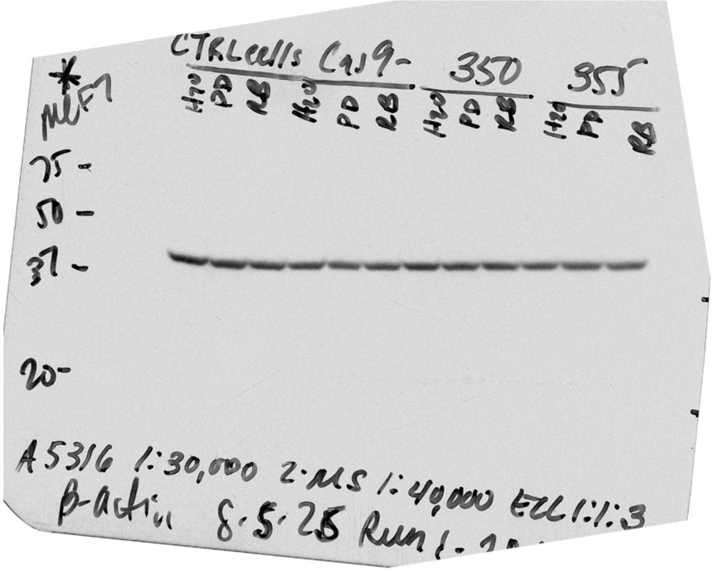

T47D7 Cells:

PFKFB3 blot: FB3 T47DpS PFKFB3CRISPR350-355 PD-RB Run1 7-31-25.tif

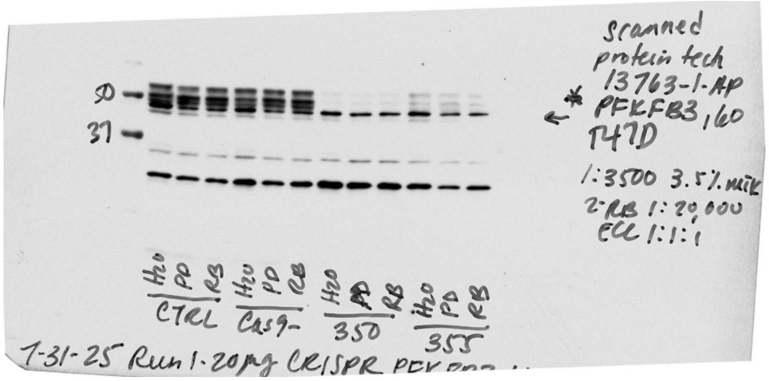

Actin blot: bActin T47DpS PFKFB3CRISPR350-355 PD-RB Run1 8-5-25.tif

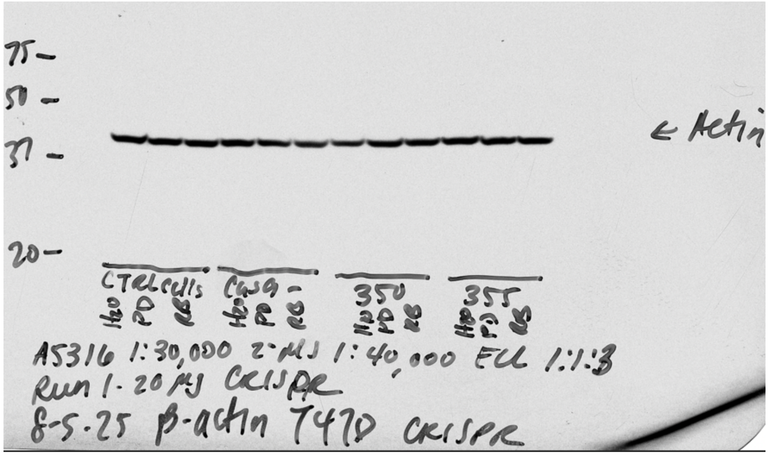

Supplement: Supplementary file 1 [file 13058_2026_2336_MOESM1_ESM.zip › Western Blots Figure 3 original scans FN.pdf]
